# The phosphoinositide signature guides the final step of plant cytokinesis

**DOI:** 10.1126/sciadv.adf7532

**Published:** 2023-07-19

**Authors:** Alexis Lebecq, Camila Goldy, Aurélie Fangain, Elsa Gascon, Katia Belcram, Martine Pastuglia, David Bouchez, Marie-Cécile Caillaud

**Affiliations:** ^1^ Laboratoire Reproduction et Développement des Plantes, Université de Lyon, ENS de Lyon, UCB Lyon 1, CNRS, INRAe, F-69342 Lyon, France.; ^2^ Université Paris-Saclay, INRAE, AgroParisTech, Institut Jean-Pierre Bourgin (IJPB), 78000 Versailles, France.

## Abstract

Plant cytokinesis, which fundamentally differs from that in animals, requires the outward expansion of a plasma membrane precursor named the cell plate. How the transition from a cell plate to a plasma membrane occurs remains poorly understood. Here, we report that the acquisition of plasma membrane identity occurs through lateral patterning of the phosphatidylinositol 4,5-bisphosphate PI(4,5)P_2_ at the newly formed cell plate membrane. There, the phosphoinositide phosphatase SAC9 emerges as a key regulator, colocalizing with and regulating the function of the microtubule-associated protein MAP65-3 at the cell plate leading zone. In *sac9-3* mutant, the polar distribution of PI(4,5)P_2_ at the cell plate is altered, leading to ectopic recruitment of the cytokinesis apparatus and formation of an additional cell plate insertion site. We propose that at the cell plate, SAC9 drives the depletion of PI(4,5)P_2_, which acts as a polar cue to spatially separate cell plate expansion from the acquisition of plasma membrane identity during final step of cytokinesis.

## Teaser

The phosphoinositide PI(4,5)P_2_ acts as a hallmark to guide the final step of plant cell division.

## INTRODUCTION

**Fig. 1.**
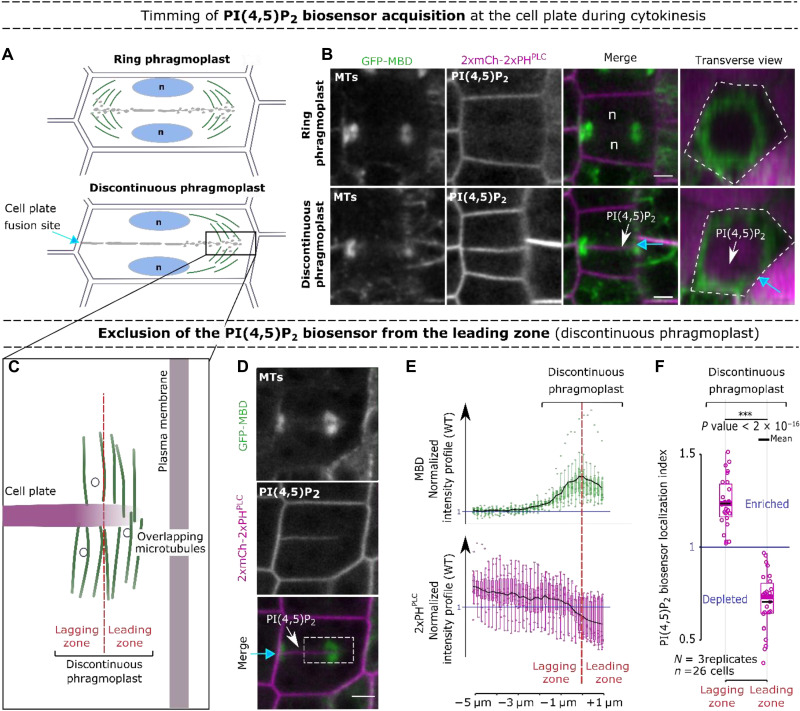
PI(4,5)P_2_ is recruited at the cell plate after its unilateral attachment. (**A**) Representation of the unattached cell plate showing a ring phragmoplast (top) and the unilaterally attached cell plate corresponding to a discontinuous phragmoplast (bottom). (**B**) Confocal images with a root tracking system (*33*) of 2xmCh-2xPH^PLC^ with the microtubule reporter line GFP fused to the microtubule-binding domain of microtubule-associated protein 4 (GFP-MBD) during the two steps represented in (A). Single longitudinal sections for each fluorescent channel and a transverse section of the merge channels are presented (0.7 μm between optical sections). Dotted line, cell contour. (**C**) Representation of the expanding edge of a discontinuous phragmoplast. (**D**) Images of 2xmCh-2xPH^PLC^ and GFP-MBD at the unilaterally attached cell plate. (**E**) Normalized intensity profiles at 6 μm along the discontinuous phragmoplast [region of interest (ROI)]. (**F**) PI(4,5)P_2_ biosensor localization index (normalized by the cell plate intensity) on the phragmoplast lagging/leading zones. In the plots, middle horizontal bars represent the median, while the bottom and top of each box represent the 25th and 75th percentiles, respectively. At most, the whiskers extend to 1.5 times the interquartile range (IQR), excluding data beyond. For range of value under 1.5 IQR, whiskers represent the range of maximum and minimum values. Results of the statistical analysis (shown in table S2) are presented (*N* = number of replicates, *n* = number of cells). White arrow, PI(4,5)P_2_ biosensor appearance; blue arrow, cell plate fusion site; red dotted line, separation leading and lagging zone; dotted lines, ROI; n, nucleus. Scale bars, 5 μm.

Cytokinesis is a key cellular process that allows the compartmentalization of living beings into cells, a necessary step for cellular heterogeneity (morphogenesis and differentiation), and in turn for proprioception (mechanical and biochemical cues channeling organ shape). Plant cytokinesis differs from the unfurrowing cytokinesis observed in animals and involves the building of a plant-specific cytoskeletal array named the phragmoplast, which, by outward growth, drives the separation of the mother cell into daughter cells (*1*). This process requires highly polarized membrane trafficking toward the phragmoplast midzone for the deposition of a transitory membrane structure named the cell plate (*2*). There, overlapping antiparallel microtubules at the outer phragmoplast edge (leading zone, Fig. 1A) drive cell plate expansion toward the cell periphery, while microtubules are disassembled in the inner phragmoplast region (lagging zone). In this process, the most likely microtubule cross-linking factors are proteins in the microtubule-associated protein 65 (MAP65)/Anaphase Spindle Elongation1/Protein Regulator of Cytokinesis1 family and members of the Kinesin-12 subfamily (*3*, *4*). Upon cell plate attachment, a number of changes in cell plate properties and composition occur (*5*–*7*), suggesting that the leading zone of the phragmoplast might play a role in cytokinesis termination (*8*). The nature of this role remains elusive, and the molecular players regulating the transition between cell plate to plasma membrane identity are poorly understood.

Recent evidence indicates a specific anionic lipid signature for the plant plasma membrane (*9*). This landmark is controlled by phosphoinositide metabolism, in particular enzymes such as phosphatases and kinases that locally interconvert the different pools of phosphoinositides (*10*). At the plasma membrane, phosphatidylinositol 4,5-bisphosphate [PI(4,5)P_2_] is enriched, whereas PI(4,5)P_2_ is excluded from the endocytic pathway (*11*, *12*). This spatial distribution of PI(4,5)P_2_ allows the polar recruitment of proteins to orchestrate processes such as membrane trafficking (*13*–*15*) and cytoskeleton remodeling (*16*–*18*). During cytokinesis, while most anionic lipids accumulate at the cell plate from its inception, PI(4,5)P_2_ remains absent (*19*, *20*). Yet, how and when the cell plate acquires a new membrane identity to become a PI(4,5)P_2_-enriched plasma membrane remains unknown.

Here, we report that PI(4,5)P_2_ enrichment at the cell plate occurs upon unilateral attachment to the maternal membrane. At this point, PI(4,5)P_2_ remains absent from the leading edge of the phragmoplast at the nonattached side of the phragmoplast, suggesting an active mechanism preventing further passive diffusion. The absence of PI(4,5)P_2_ at the leading edge correlates with the enrichment of the putative PI(4,5)P_2_ phosphatase SUPPRESSION OF ACTIN 9 (SAC9). Loss of *sac9* leads to the ectopic accumulation of PI(4,5)P_2_ at the phragmoplast leading edge, which correlates with MAP65-3 mislocalization and abnormal cell plate branching starting from the MAP65-3 inner domain at the cell plate. We propose a model in which the absence of PI(4,5)P_2_ (or the presence of SAC9) acts as a polar cue to guide the leading zone of the phragmoplast during cell plate attachment.

## RESULTS

We investigated how PI(4,5)P_2_ membrane signature is acquired during cytokinesis, using the *Arabidopsis* root meristem as a model. Live cell imaging in three-dimensional (3D) and time confirmed that during cytokinesis, PI(4,5)P_2_ biosensors (mCit-TUBBYc or 2xmCH-PH^PLC^) (*11*) are absent from the expanding cell plate until its unilateral attachment to the mother cell (Fig. 1, A and B, and figs. S1 and S2). These observations suggest that PI(4,5)P_2_ enrichment at the maturing cell plate (Fig. 1B, white arrow) probably arises by passive diffusion from the highly fluid lateral plasma membrane of the mother cell.

At this stage, cell plate attachment (blue arrow) is not synchronous along its periphery, allowing for comparison of attached and nonattached regions of the same cell plate (Fig. 1A). At the leading zone of the partially attached cell plate, where the phragmoplast was still present, a statistically significant decrease of PI(4,5)P_2_ biosensor fluorescence (index <1) was observed (Fig. 1, C to F, and Figs. S1 and S3) suggesting an active mechanism preventing PI(4,5)P_2_ accumulation to the newly formed membrane domain at the phragmoplast leading zone.

**Fig. 2.**
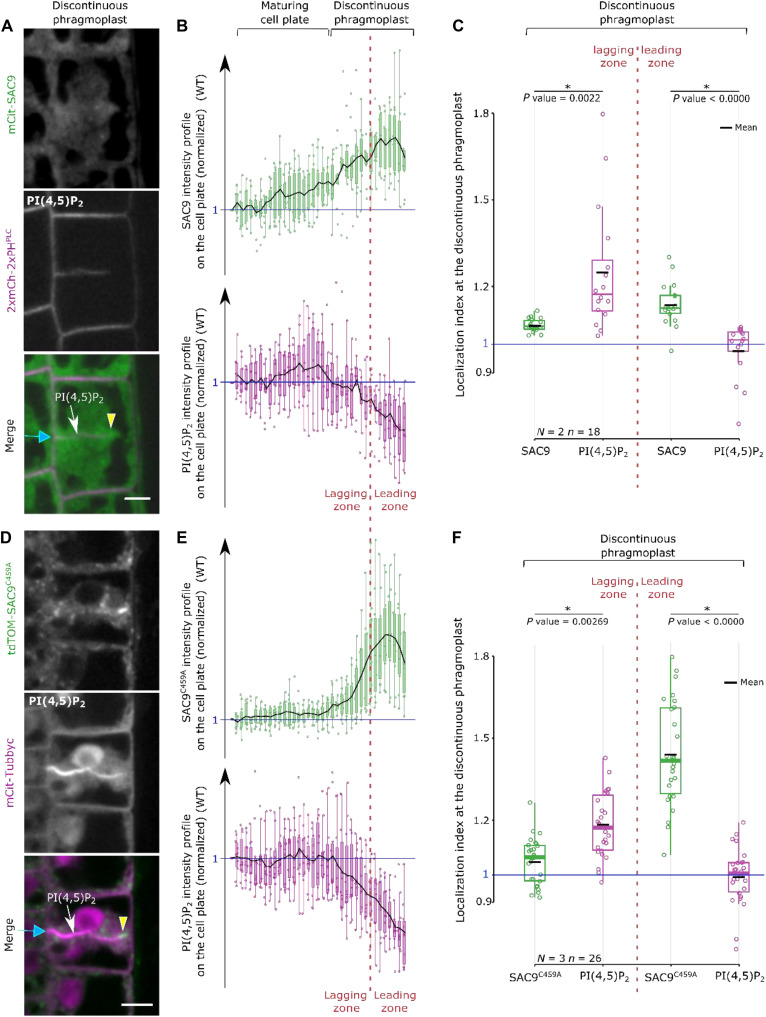
SAC9 and the PI(4,5)P_2_ are mutually co-excluded at the cell plate. (**A**) Images of mCit-SAC9 with 2xmCh-2xPH^PLC^ when the cell plate is unilaterally attached. (**B**) Normalized intensity profiles corresponding to (A). (**C**) Quantification of the localization index corresponding to (A). (**D**) Images of tdTOM-SAC9^C459A^ with mCit-Tubbyc when the cell plate is unilaterally attached. (**E**) Normalized intensity profiles corresponding to (D). (**F**) Quantification of the localization index corresponding to (D). Blue arrow, cell plate fusion site; yellow arrowhead, SAC9 enrichment; red dotted line, separation leading and lagging zone; n, nucleus. Scale bar, 5 μm.

**Fig. 3.**
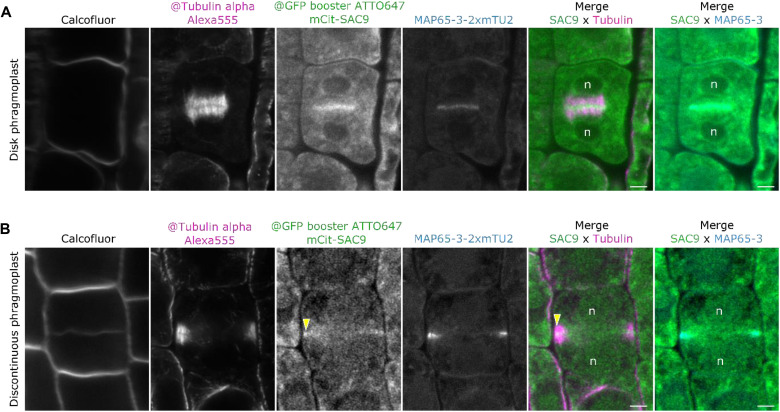
Super-resolution microscopy reveals the colocalization of SAC9 with MAP653 at the leading edges of the cell plate. (**A** and **B**) From left to right: cell wall marker, calcofluor, super-resolution microtubules revealed with @tubulin alpha antibodies (alexa555 secondary antibodies) with mCit-SAC9 revealed by GFP-booster ATTO647 antibody accompanied by MAP65-3-2xmTU2 preserved fluorescence. Two phragmoplast stages are presented: the disk phragmoplast (A) and discontinuous phragmoplast (B). Yellow arrowhead, SAC9 enrichment; n, nucleus. Scale bar, 5 μm.

To identify the molecular components driving the exclusion of PI(4,5)P_2_ from the leading zone of the expanding cell plate, we analyzed the subcellular localization of the plant-specific enzyme SAC9, which participates in the restriction of PI(4,5)P_2_ at the plasma membrane during endocytosis (*12*). We observed that a mCit-SAC9 fusion is enriched at the phragmoplast leading zone (index >1, Fig. 2, A to C, yellow arrow; figs. S4 and S5), suggesting a role for this enzyme in PI(4,5)P_2_ dephosphorylation. To test the direct relationship between the function of SAC9 and the patterning of its substrate PI(4,5)P_2_, we mutated the cysteine in the conserved C-x(5)-R-[T/S] catalytic motif found in all SAC domain-containing phosphoinositide phosphatase (SAC9^C459A^) (*12*). We analyzed the localization of mCit-SAC9^C459A^ likely catalytically inactive and not able to rescue the mutant phenotype (*11*). We first confirmed that mCit-SAC9 and tdTOM-SAC9^C459A^ colocalized at the leading edge of the cell plate (fig. S6, A, and B). Like mCit-SAC9, tdTOM-SAC9^C459A^ was enriched at the leading zone while PI(4,5)P_2_ was depleted (Fig. 2, D to F, and fig. S6, C and D). When SAC9 or the allelic variant SAC9^C459A^ was visualized together with the microtubule-associated protein MAP65-3 specifically localizing to the phragmoplast midzone (*21*), a spatial and temporal colocalization at the cell plate leading zone was observed (Fig. 3; fig. S6, E to H; and movie S1), a feature shared by only a few proteins (*4*, *8*).

**Fig. 4.**
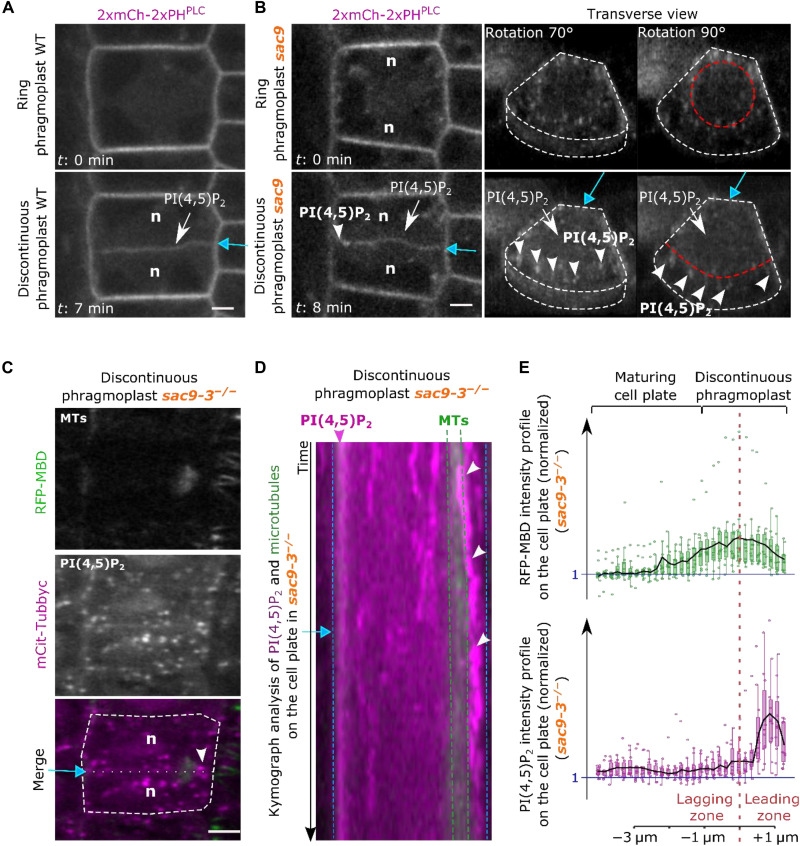
In *sac93*, PI(4,5)P_2_ pool precedes the phragmoplast leading edge during late cytokinesis. (**A**) Images extracted from time-lapse imaging in WT of 2xmCh-2xPH^PLC^ at two cytokinetic steps: ring (top) and discontinuous (bottom) phragmoplast stages. (**B**) Images extracted from time-lapse imaging in *sac9-3* of 2xmCh-2xPH^PLC^ at two cytokinetic steps: ring (top) and discontinuous (bottom) phragmoplast stages. On the right panel are presented two transverse views of 2xmCh-2xPH^PLC^ for both stages in *sac9-3* (0.3 μm between optical sections). (**C**) Images of mCit-Tubbyc with RFP-MBD in *sac9-3.* (**D**) Kymograph representation of PI(4,5)P_2_ and MTs at the cell plate during the discontinuous phase in *sac9-3* [related to (C)]. Images were taken every 5 s for a total duration of 5 min. (**E**) Normalized intensity profiles corresponding to (C). White arrow, PI(4,5)P_2_ biosensor appearance; white arrowhead, abnormal PI4,5)P_2_ enrichment at the leading zone in *sac9-3*; purple arrowhead, PI(4,5)P_2_ biosensor continuous enrichment at the cell plate–plasma membrane junction; white dotted line, cell contour; red dotted line, separation leading and lagging zone; green dotted line, delimitation of the phragmoplast; blue arrow, cell plate fusion site; n, nucleus. Scale bars, 5 μm.

Our results suggest that active restriction of PI(4,5)P_2_ from the leading zone during cell plate attachment is mediated through the enzymatic activity of SAC9. If true, the absence of SAC9 should affect PI(4,5)P_2_ distribution at the cell plate. To test this hypothesis, we assessed PI(4,5)P_2_ distribution at the cell plate in the *sac9-3* loss of function mutant. In *sac9-3*, PI(4,5)P_2_ biosensors are no longer restricted to the plasma membrane and also ectopically accumulate in endosomes coming from the endocytic pathway (*12*). After unilateral attachment of the cell plate, PI(4,5)P_2_ biosensor enrichment at the maturing cell plate was indeed observed both in the wild type (WT) and in the *sac9-3* mutant. In *sac9-3*, we additionally observed an ectopic accumulation of PI(4,5)P_2_ biosensors at the leading zone (white arrowhead), probably being incorporated from cytokinetic vesicles (index >1, Figs. 3F and 4A and figs. S6 and S7). Observations in three dimensions revealed that a PI(4,5)P_2_ pool was constantly observed on the leading edge of the phragmoplast in *sac9-3* (Fig. 4E). During late cytokinesis in *sac9-3*, the PI(4,5)P_2_ pool upfront of the phragmoplast eventually was incorporated to the attached cell plate (Fig. 4E and movie S2). Moreover, the ectopic accumulation of PI(4,5)P_2_ biosensor observed in *sac9-3* spatially correlates with the accumulation of the nonfunctional SAC9^C459A^ at the leading edges of the cell plate, pointing out toward a role of the enzyme in the dephosphorylation of the PI(4,5)P_2_ at endosomes (figs. S6 and S7). The abnormal PI(4,5)P_2_ pattern in the *sac9-3* mutant is consistent with the idea that SAC9 restricts PI(4,5)P_2_ and prevents its premature enrichment in the cell plate leading zone (fig. S8). The fact that endosomes rich in PI(4,5)P_2_ accumulate at the cell plate leading edge in *sac9-3* cells also suggests that such abnormal PI(4,5)P_2_ signature on endosomes originating from endocytosis prevents their fusion with the expanding cell plate (fig. S8). The contribution of the endocytic traffic to plant cytokinesis is still under debate and future research might help us to better characterize this phenomenon. To rule out that the phosphatidylinositol-4-phosphate (PI4P) production through the PI(4,5)P_2_ conversion by SAC9 is also involved, we quantified the accumulation of the PI4P biosensor 2xmCH-2xPH^FAPP1^ at the cell plate. No significative difference was observed between the WT and the *sac9-3* mutant, suggesting that only the PI(4,5)P_2_ patterning is impaired in the absence of SAC9 (fig. S9).

**Fig. 5.**
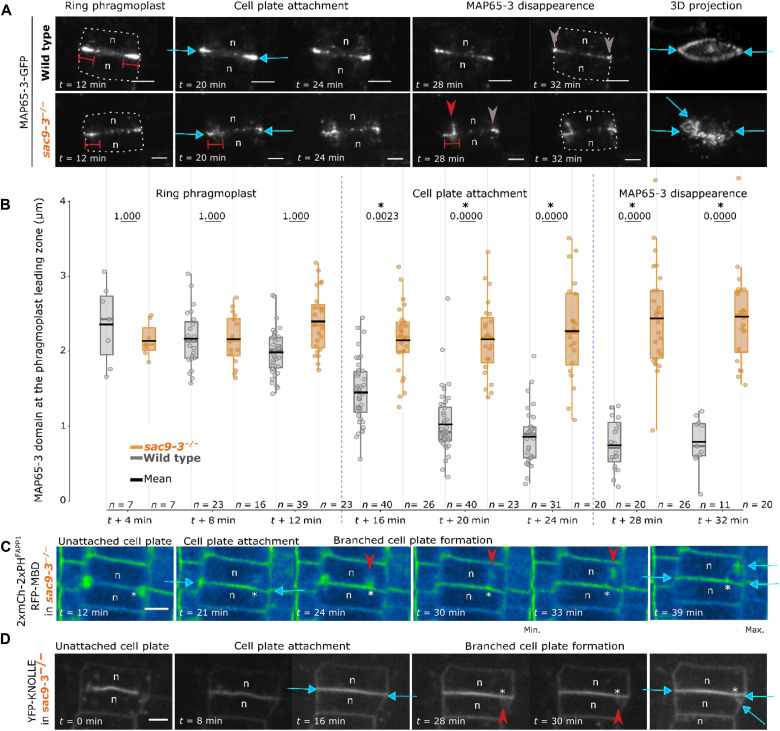
De novo recruitment of the cytokinesis apparatus in *sac9-3*. (**A**) Image series of MAP65-3–GFP in WT (top) or *sac9-3* (of the bottom) every 2 min for 2 hours. Time is given relative to “disk”-"ring” phragmoplast transition (0 min) and cell plate attachment (14 min). Finals images represent a 3D view of time = 32 min. (**B**) Comparison of MAP65-3–GFP domain length in WT versus *sac9-3*. In the plots, middle horizontal bars represent the median, while the bottom and top of each box represent the 25th and 75th percentiles, respectively. At most, the whiskers extend to 1.5 times the interquartile range, excluding data beyond. For the range of values under 1.5 IQR, whiskers represent the range of maximum and minimum values. Results of the statistical analysis (shown in table S4) are presented (*n* = number of cells). (**C**) Z projection of 2xmCherry-2xPH^FAPP1^ and RFP-MBD in *sac9-3* over time. Here, the signal is color-coded in green fire blue (see scale bar on the right). The signal corresponding to microtubules and the one corresponding to the membrane are not distinguished). Note that here, 2xmCh-2xPH^FAPP1^ is used at the membrane marker (plasma membrane and cell plate). (**D**) Image series of YFP-KNOLLE in *sac9-3*. Gray arrow, MAP65-3 localization after cytokinesis; double red bars, distance labeled by GFP–MAP65-3; red arrowhead, branch emergence in *sac9-3*; blue arrow, cell plate fusion site; asterisk, position of the branch’s emergence at the cell plate; n, nucleus. Scale bars, 5 μm.

Next, we investigated the functional relevance of the SAC9-dependent PI(4,5)P_2_ pattern at the cell plate for cytokinesis. Because phragmoplast organization is regulated by MAP65-3 (*22*–*25*) that colocalizes with SAC9 (Fig. 3, A and B, and fig. S6, E and F), we addressed the localization of MAP65-3 in the absence of SAC9. During phragmoplast expansion, MAP65-3–GFP (green fluorescent protein) dynamic was similar (*P* value: 0.00), with a constant MAP65-3 domain of about 2 μm (Fig. 5, A and B). In WT, the MAP65-3 domain gradually decreased in later stages to eventually disappear (Fig. 5, A and B). In contrast, in *sac9-3* mutant cells, the MAP65-3–GFP domain remained unchanged, at a constant distance of 2.28 μm from the phragmoplast leading edge (Fig. 5, A and B, and movies S3 to S5), suggesting a failure to transition to the final stage of cytokinesis in the absence of SAC9.

During defective cytokinesis in *sac9-3*, the persistence of MAP65-3 on the cell plate leading zone always correlated with the emergence of a “branch” from the MAP65-3–GFP inner domain (Fig. 5A, red arrow, and fig. S10A). Eventually, the branch labeled by MAP65-3–GFP fused to the maternal membrane at an ectopic site (Fig. 5A and movies S4 and S5). On the branch, MAP65-3–GFP behaved as it did in the main phragmoplast, being progressively restricted to the leading zone and ultimately disappearing upon attachment (Fig. 5A and movies S4 and S5). Quantification of the width/length of the expanding phragmoplast before the first attachment, using RFP fused to the microtubule binding domain of microtubule associated protein 4 (RFP-MBD) or LifeAct-YFPv as a readout for microtubules and actin respectively, showed no differences between WT and *sac9-3* (fig. S10, A to G). After cell plate attachment, the microtubule phragmoplast disassembled from the main cell plate in *sac9-3* as it did in the WT plant (Fig. 5C and fig. S10, H to J). However, in the branching events observed in *sac9-3*, a pool of microtubules re-engaged as a phragmoplast-like structure on the fully expanded cell plate, at ~2 to 3 μm from the cell plate fusion site, corresponding to the inner face of the MAP65-3–GFP domain (*N* = 3, red arrow, Fig. 5C and fig. S10J). This finding supports the model in which the phosphoinositide signature coordinates cell plate and microtubule integrity during late cytokinesis.

We tested the genetic interaction between SAC9 and an upstream regulator of MAP65-3, the mitogen-activated protein kinase MPK4 (*26*). MPK4 interacts genetically and physically with the phosphoinositide kinase PI4Kβ1 to control microtubule stability during phragmoplast expansion (*18*). We observed that the *sac9-3,mpk4-2* double mutant is phenotypically indistinguishable from *sac9-3* up to 14 days after germination. Later, *sac9-3,mpk4-2* plants failed to grow and eventually died before giving a progeny (fig. S11). Cell plate branching was not observed in *mpk4-*2 or *map65-3/dyc283*, probably because in these mutants, phragmoplast expansion stops before the complete final attachment, leading to the formation of characteristic cell wall stubs (*21*, *26*, *27*). In the *sac9-3,mpk4* double mutant, the presence of both cell wall stubs characteristics of the *mpk4-2* mutant and of branched cell plates characteristics of the *sac9-3* mutation were detected (fig. S11). Intriguingly, in *mpk4-2* plant complemented with a constitutively active MPK4 kinase (*MPK4^D198G/E202A^*) (*28*), cell plate branching resembling the one observed in *sac9-3* was observed (around 1.3 defects per root; fig. S11) suggesting that MPK4 activity may be involved in the cell plate branching phenotype observed in the absence of SAC9. Whether or not this is directly linked to the function of MAP65-3 still needs to be addressed.

To test our conclusion, we modified the system in a different way. Because the ultimate effector the microtubule cytoskeleton is the main component of the phragmoplast, we reasoned that microtubule disruption could lead to a similar phenotype. We then tested the sensitivity of *sac9-3* to mild perturbations of the cytoskeleton using pharmacological treatments (fig. S12). In plants treated with 1 μM Taxol (fig. S12, A and B) or 1.5 μM propyzamide (fig. S12, C and D), root growth reduction was similar in *sac9-3* and in the WT. At the subcellular level, WT plants grown on 1 μM Taxol or 1.5 μM propyzamide did not display detectable cytokinesis defects (fig. S12, B and D). At higher concentrations (25 and 50 μM respectively for 24 hours) cell plate branching was detected in WT, as well as after treatment with 20 μM chlorpropham (fig. S13, A and B). Perturbation of the actin cytoskeleton using 0.1 μM latrunculin B for 24 hours did not induce the formation of branched cell plate, which might suggest that this phenomenon does not require directly actin cytoskeleton integrity (figs. S12, E and F, and S13B). In contrast to *sac9-3*, cell plate branching induced by microtubule drugs often emerged before the first attachment (fig. S13A), highlighting a broader effect of the drugs on phragmoplast expansion. In WT plants treated with 25 μM Taxol, MAP65-3–GFP decorated microtubules of the branched phragmoplast, whereas in *sac9-3*, localization of MAP65-3–GFP was restricted to the phragmoplast midzone (Fig. 5A and fig. S13C). However, in all cases, enrichment in MAP65-3–GFP was observed at the initiation site of the branch (Fig. 5A and fig. S13, B and C). On the basis of our findings, we conclude that the phenotype observed in *sac9-3* might not result from defects in microtubule dynamics and phragmoplast expansion per se, but rather from defective perception of cell plate attachment, resulting in ectopic reactivation of the phragmoplast.

**Fig. 6.**
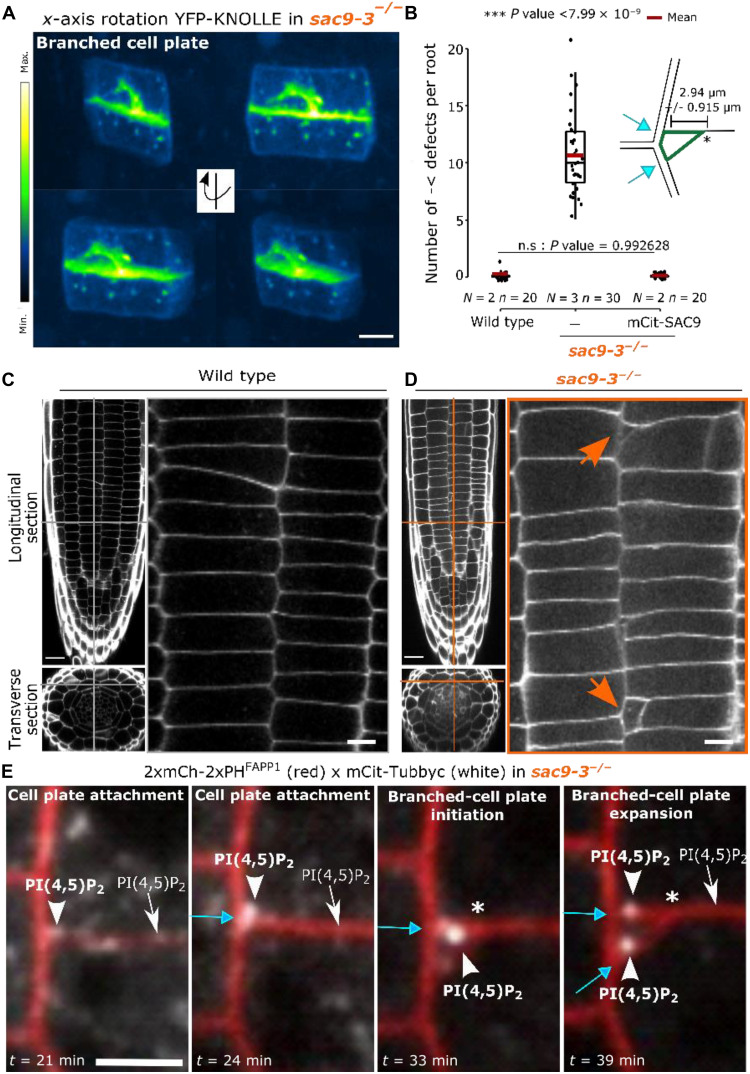
Ectopic membrane domain formed in the absence of SAC9 has a cell plate identity and leads to cell wall defects. (**A**) 3D rotation of YFP-KNOLLE in *sac9-3* during defective cytokinesis. Here, the signal is color-coded in green fire blue (see scale bar). Scale bars, 5 μm. (**B**) Quantification of the number of defects. On the top right, representation of branched cell wall defects (green) with key topological elements listed such as the distance between the branched cell wall and lateral cell wall (double arrow), and cell plate insertion site (blue arrow). In the plots, middle horizontal bars represent the median, while the bottom and top of each box represent the 25th and 75th percentiles, respectively. At most, the whiskers extend to 1.5 times the interquartile range, excluding data beyond. For a range of values under 1.5 IQR, whiskers represent the range of maximum and minimum values. Results of the statistical analysis (shown in table S5) are presented (*N* = number of replicates, *n* = number of cells). (**C** and **D**) Z-stack images of calcofluor-stained fixed roots (7-day-olds) for WT (C) and *sac9-3* (D). Orange lines, the position of the section. Left: Longitudinal section (top: scale bars, 20 μm), and transverse section (bottom). Right: A crop of the root cortex (scale bars, 5 μm). (**E**) Time series of mCit-Tubbyc and 2xmCh-2PH^FAPP1^ at the end of the cytokinesis in *sac9-3* (bars, 5 μm). Here, 2xmCh-2xPH^FAPP1^ is used as a marker for membranes (plasma membrane and cell plate). n, nucleus; white asterisk, position of the branch’s emergence at the cell plate; orange arrow, cell wall defects; blue arrow, cell plate fusion site; white arrowhead, PI(4,5)P_2_ biosensor enrichment observed in *sac9-3*. Scale bars, 5 μm.

Using KNOLLE localization as a readout for cell plate fusion defects (*8*, *29*, *30*), we showed that in contrast to what was previously reported for mutations impairing membrane fusion, no dispersed vesicular localization was observed for YFP-KNOLLE in *sac9-3* (fig. S14). Moreover, YFP-KNOLLE decorated the branching cell plate, indicating that this ectopic membrane domain has a cell plate identity (Figs. 5D and 6A). Abnormal branching represented 4% of the total apicobasal cells walls (~11 cells per root, *n* = 30 roots, *N* = 3 replicates), which is comparable to what is often observed for mutants impaired in cytokinesis (*18*, *31*). Cell wall defects observed in *sac9-3* were exclusively positioned at the crosswall in proximity to the mother cell lateral wall, with an intersection relatively constant at 2.93 μm ± 0.91 SD (Fig. 6, B to D, and movies S8 and S9), reminiscent of the distance labeled by MAP65-3–GFP and mCit-SAC9 at the phragmoplast leading zone in WT (Fig. 3; Fig. 6B, white asterisk; and fig. S5).

To determine whether the phenotype observed was due to mispositioning of the cell division site, we analyzed the position of the preprophase band using our MAP65-3 marker as a readout (*27*, *32*). We imaged MAP65-3–GFP in *sac9-3* dividing cells, from early mitosis to late cytokinesis, using our root tracking system (*33*). While the first attachment of the cell plate indeed corresponds to the position previously occupied by the preprophase band, no MAP65-3–GFP signal was observed at ectopic attachment sites in *sac9-3* (fig. S15A). According to the existing model, the cell plate attachment site within the cortical division zone is facilitated by a set of specialized proteins (**2**). We assessed the localization of the cortical division zone marker PHRAGMOPLAST ORIENTING KINESIN 1 (POK1) (*34*). In the absence of SAC9, YFP-POK1 was observed at the cortical division zone in two distinct spots (blue arrows) corresponding to the first attachment site but also at the abnormal second cell plate attachment site observed in *sac9-3* defective cytokinesis (fig. S15B). This finding suggests that in the absence of SAC9, the cell plate extension and its guidance are reactivated leading to the formation of an abnormal branched cell plate.

To test the temporal specificity of this phenotype, we reasoned that a transient rescue may be sufficient. By engineering a construct fusing SAC9 to the expression, localization, and degradation signals of MAP65-3 (*24*) we were able to mitotically express SAC9, target it to the cell plate, and degrade it after mitosis (fig. S16). Using this construct, we showed that expressing *SAC9* during mitosis was sufficient to rescue the *sac9-3* cytokinesis defect as well as part of its dwarf phenotype, highlighting the importance of SAC9 function during cell division. However, we cannot exclude the possibility that the mitotic expression of SAC9 may be sufficient to restore PI(4,5)P_2_ distribution at the plasma membrane in later stages, thereby complementing aspects unrelated to cell division as well.

Concomitantly to the cell plate-branching in *sac9-3*, a specific distribution of PI(4,5)P_2_ biosensor was observed, with a burst (i) at the cell division site where the cell plate attached, (ii) on the cell plate, at ~3 μm where the branching was initiated, (iii) and at the ectopic cell division site where the cell plate-branched was inserted (*N* = 5; white arrowhead, Fig. 6E, fig. S17, and movie S10). Moreover, mCit-*SAC9^C459A^* was not able to rescue the phenotype observed (fig. S18). Together, our findings suggest that the phosphatase activity of SAC9 [and thereby its role in the PI(4,5)P_2_ repartition] is critical to coordinate cell plate attachment through MAP65-3 behavior.

## DISCUSSION

Cytokinesis in multicellular organisms dictates how cells are organized in a tissue, their identities, and their functions. At the heart of cytokinesis, the coordination between the cytoskeleton and the vesicle trafficking is of paramount importance as it drives the separation of the mother cell into daughter cells. In this context, an extensive number of studies on the role of proteins implicated in the cytoskeleton’s remodeling and trafficking have been reported (*35*). Yet, the mechanism by which the final step of cell division occurs remains unclear.

Here, we took an unexplored angle, looking at the role of membrane lipids during the final steps of cytokinesis. Using Arabidopsis as a model, we showed that the anionic lipid signature controls cytokinesis in plants. This analysis was possible thanks to the identification and characterization of a class of mutants in which the last step of cytokinesis is not perceived. In this mutant, which is impaired in phosphoinositide metabolism (*12*), defects during the final step of cytokinesis provoke abnormal multiple–cell division sites in a given cell. The branching of the cell plate is to our knowledge a rare phenomenon that has been only observed after mild perturbation of the microtubule cytoskeleton (this study) or in the conditional mutant *mor1-1* (*36*). This mutant, impaired in a member of the XMAP215 family of microtubule-associated proteins, displays at restrictive temperature branched cell plates but also incomplete, asymmetric, wandering cell plates (*36*). The stereotyped branched cell plates observed in the absence of SAC9 appear therefore more specific than a general disruption of the microtubule cytoskeleton. We wondered whether the aberrant branching observed in *sac9* preferentially occurs at the unattached phragmoplast side after unilateral attachment. However, because of the technical limitations of live cell imaging in four dimensions in the native tissue, we cannot answer this point in a quantitative manner.

We showed that the cell plate branching observed in the absence of SAC9 does not result from a PPB positioning defect but is rather linked to the formation of a secondary cortical division zone where YFP-POK1 is enriched. We showed that after pharmacological perturbation of microtubules arrays in the sac*9-3* mutant, the branching of the cell plate always coincided with the enrichment in MAP65-3–GFP at the site of initiation. Because of technical limitations, we are not able to know whether the signal observed for POK1 at the abnormal second cell plate attachment site appears early during mitosis or later during the cell plate branching. If POK1 was present early during division at two sites, then the first attachment is *sac9-3* should occur at 50% in one site and 50% at the other site, where POK1 is accumulating. This would lead to a phenotype characterized by tilted cell plates (*37*, *38*). Because the quantification of the arrangement of cell walls perpendicular to the growth axis shows no difference between *sac9-3* and WT (fig. S19), we hypothesize that POK1 accumulation at the ectopic cortical division zone is more likely to appear in late cytokinesis.

We found that SAC9 progressively disappears from the fully attached cell plate upon its unilateral attachment to the mother cell. Consequently, SAC9 does not localize to the plasma membrane in the resulting interphasic cells (*12*). Hence, SAC9 and its substrate are mutually co-excluded spatially. Our previous work suggests that SAC9 function is more likely restricted to endosomes emerging from the plasma membrane during endocytosis than directly at the plasma membrane (*12*). We, therefore, do not expect the PI(4,5)P_2_ level to increase at the plasma membrane or at the maturing zone of the cell plate. We rather think that SAC9 mutation is most likely affecting the ratio between the PI(4,5)P_2_ at the plasma membrane and the internalized PI(4,5)P_2_. This is why the PI(4,5)P_2_ biosensors that are expressed at a similar level in WT and *sac9-3* are mainly labeling the pool of PI(4,5)P_2_ in endosomes rather than the PI(4,5)P_2_ pool present at the plasma membrane (*12*). This phenomenon is extreme for the TUBBYc biosensor, but the use of two independent biosensors to detect the PI(4,5)P_2_ used in this study allows us to observe a consensus localization at the leading edge of the cell plate for the PI(4,5)P_2_ biosensors in the absence of SAC9.

To test the direct relationship between the function of SAC9 and the patterning of its substrate the PI(4,5)P_2_, we generated a catalytic dead version of SAC9 (*12*). Here, we showed that while *sac9-3* is complemented with *SAC9pro:mCit-SAC9*, the expression of *SAC9pro:mCit-SAC9^C459A^* was not sufficient to rescue the cytokinesis defects observed in *sac9-3*. We previously confirmed that mCit-SAC9^C459A^ fusion was stable and accumulated to a similar extent as mCit-SAC9 (*12*). Because the putative catalytic cysteine, C459, is required for SAC9 function during cytokinesis, we can conclude that the phosphatase activity of SAC9 is participating in the observed phenotype. Given that phosphoinositide’s metabolism is highly intricate, we recognize that it is difficult to fully untangle the specific involvement of each lipid in the observed phenotypes. Moreover, SAC9 may carry specific functions outside of its catalytic activity, and therefore, the phenotype observed could be due to other factors, such as the recruitment of protein partners.

Because both SAC9 and the allelic variant mutated in the catalytic site are both recruited to the leading edge of the cell plate and to membranes in interphasic cells, we speculate that SAC9 targeting to membranes is regulated by interaction with a protein partner rather than by its substrate. Because of the colocalization between SAC9 and MAP65-3 and the phenotype observed in constitutively active MPK4, it is tempting to speculate that these proteins might be interacting. However, because SAC9 is recruited to membranes in both interphasic and dividing cells, we rather suspect protein partners not specifically expressed during mitosis. Hence, Ras-associated binding (RAB), small guanosine triphosphatases regulating protein transport along the endocytic and exocytic pathways are good candidates to regulate SAC9 localization. This phenomenon is well known in animal cells, where the functional homologs of SAC9, the PI(4,5)P_2_ 5-phosphatase OCRL is recruited to membranes during cytokinesis via its interaction with RAB35 (*39*).

Numerous pieces of evidence coming from the animal literature highlight the connection between the PI(4,5)P_2_/PI4P balance, to the function and the recruitment of actin-binding proteins and actin nucleators (*40*). In plants, little is known about the role of the actin cytoskeleton during cytokinesis, and the link with the phosphoinositide metabolism in the context of mitosis is also hypothetical. The increased number of defects observed in the *sac9-3* mutant after disruption of the actin cytoskeleton (fig. S12F) suggests a cross-talk between the phosphoinositide metabolism and the actin cytoskeleton during cell plate attachment. Whether or not the accumulation of PI(4,5)P_2_ endosomes promotes F-actin polymerization and perturb polarity/cell plate behavior in the sac9-3 remains to be addressed in further detail in the future.

Using PI(4,5)P_2_ biosensors, we observed that PI(4,5)P_2_ is depleted from the leading edge of the cell plate, where SAC9 restricts its patterning. Passive diffusion of PI(4,5)P_2_ or more generally high PI(4,5)P_2_ may induce a switch in microtubule dynamics to stop phragmoplast expansion. It was recently shown that upon PI(4,5)P_2_ inactivation using an inducible PI(4,5)P_2_ depletion system, highly anisotropic transverse cortical microtubules were replaced by randomly arranged arrays, suggesting a function of PI(4,5)P_2_ in the organization of microtubules in plant cells. Active exclusion of PI(4,5)P_2_ at the unattached leading end of the phragmoplast may thus be required to maintain microtubule dynamics until the successful completion of cytokinesis.

We observed that SAC9 undergoes the exact same changes in localization as MAP65-3, first enriched on the entire cell plate (disk phragmoplast stage), then accumulated at the leading edges and absent at the center of the maturing cell plate. The fact that SAC9 accumulates at the cell plate at an early stage might suggest that PI(4,5)P_2_ accumulation is actively restricted at the expanding edge. Even though we cannot rule out that SAC9 has another function during the early phases of phragmoplast formation, the phenotype observed in *sac9* suggests that SAC9 function is required during the cell plate attachment, preventing the premature accumulation of the PI(4,5)P_2_ at the growing leading edges.

It is plausible that during cell plate attachment, SAC9 is important for vesicle fusion. In this case, the additional cell plate attachment sites could be caused by abnormal fusion of vesicles or membrane remodeling in addition to changes in the phosphoinositide signature. Yet, no dispersed vesicular localization was visible in the *sac9-3* mutant using confocal microscopy (this study) and electron microscopy (*41*), suggesting that the additional cell plate attachment sites are probably not caused by abnormal fusion of vesicles. Fusion defects during cytokinesis often result in multinucleated cell formation (*42*, *43*), which was also not observed in *sac9-3* (*41*). When we compared the already published electron micrograph of *sac9-3* (*41*) with the micrograph of mutants impaired in membrane fusion (*29*, *44*, *45*), the characteristic fusion defect phenotype is not visible in *sac9-3* while cell wall protuberances emerging from the cell plate are clearly visible (*41*). Together, our confocal imaging and already available *sac9-3* electron micrograph do not support classical defects in the vesicular fusion at the cell plate. The phenotype observed in *sac9-3* electron micrographs resembles what was reported for the microtubule-associated protein mutant *mor1–1* in which bifurcated cell plates and abnormal cell wall structures were observed (*36*), suggesting that SAC9 might function in a pathway together with components of the microtubule cytoskeleton. It might be the case that the abnormal PI(4,5) P2-containing endosome observed around the leading edge in *sac9-3* failed to be incorporated into the cell plate because of their abnormal membrane identity. Future directions should better address the fusion of the cell plate with the plasma membrane. So far, while the homotypic fusion of vesicles during cell plate expansion is well described (*46*), the mechanism allowing the fusion of the cell plate to the plasma membrane is poorly documented. To our knowledge, only one study refers to this phenomenon (*47*). In this paper, the loss of the clathrin-mediated endocytosis adaptor *TPLATE* by RNA interference (RNAi) in BY-2 cell lines leads to “anchoring” defects of the cell plate. In this case, fusion defects are associated with the massive recruitment of material/vesicles, close to the cell plate insertion site (*47*). On the basis of the current knowledge, we cannot exclude that *SAC9* plays a role during the fusion of the cell plate with the plasma membrane. Nonetheless, the phenotype observed in TPLATE RNAi cells (*47*) differs from what we observed in the absence of SAC9, which might indicate that SAC9 is important for dephosphorylating PI(4,5)P_2_ at/close to the cell plate edges rather than vesicle fusion. It is tempting to suggest that the cell plate fusion to the plasma membrane allows for the fusion of heterogeneous material as PI(4,5)P_2_ containing endosomes. If true, this may explain why cytokinesis defects appear only at this stage in *sac9-3*, and also differences are observed with microtubules destabilizing drugs. Future directions should include the identification of the molecular machinery controlling the cell plate fusion with the plasma membrane.

We propose that upon unilateral cell plate attachment to the plasma membrane, polarity domains (i.e., cell plate maturing domain versus cell plate leading edges) are formed and the phragmoplast lagging zone acts as a buffer between the PI(4,5)P_2_-enriched maturing zone and the still expanding cell plate leading edge. In the absence of SAC9, the presence of PI(4,5)P_2_ upfront of the phragmoplast might perturb the function of cytoskeleton components such as MAP65-3, provoking the reassembly of the phragmoplast apparatus on the inner side of the leading zone. An alternative model could be that the accumulation of PI(4,5)P_2_-rich endosomes close to the leading edge but disconnected from the cell plate influences cell plate growth and branching in the *sac9-3* mutant. In any case, our findings support the model by which the absence of PI(4,5)P_2_ (or the presence of SAC9) might act as a polar cue to guide the leading zone of the phragmoplast at the final step of the plant cytokinesis (fig. S20).

## MATERIALS AND METHODS

### Growth condition and plant materials

*Arabidopsis *Columbia-0 (Col-0) accession was used as a WT reference genomic background throughout this study. All transgenic lines used in this study are listed in table S1. Arabidopsis seedlings were grown in vitro on half Murashige and Skoog (½ MS) basal medium supplemented with 0.7% plant agar (pH 5.7) in continuous light conditions at 21°C. Seedlings were imaged between 5 and 7 days after germination (dag) and lastly grown in soil under long-day conditions at 21°C and 70% humidity 16-hour daylight. For drug treatments, WT and *sac9-3* seedlings were grown in vitro for 6 days and then transferred on ½ MS basal medium supplemented with 0.7% plant agar and either 20 μM chlorpropham or 25 μM Taxol, 50 μM oryzalin, 10 μM propyzamide, 5 μM latrunculin B. Images were taken after 24 hours of incubation. For root growth experiments, *sac9-3* and WT seedlings were grown, after 3 days at 4°C in the dark, on ½ MS plates supplemented with various concentrations of drugs: 1 μM Taxol, 1.5 μM propyzamide, or 0.1 μM latrunculin B for 7 days.

### Cloning and plant transformation

Cloning of MAP65-3pro::2xmTU2-MAP65-3: The MAP65-3pro (1,2 kb) was flanked with attB2R and attB3 sequences and recombined by BP gateway reaction into pDONR221 (Fw: GGGGACAACTTTGTATAGAAAAGTTGCTTACACTCTTCCCTACACAAAACCGCG; Rv: GGGGACTGCTTTTTTGTACAAACTTGCTTCGAAATGCTTAAGCCTGTAACAGG). Final destination vectors (MAP65-3pro/P5′, MAP65-3/pDON207 (*27*), 2xmTU2/pDONR-P3′) were obtained using three fragments LR recombination system (Thermo Fisher Scientific, www.thermofisher.com) using pK7m34GW destination vector (*48*).

The collection of phosphoinositide biosensors used in this study was described previously (*11*). A tandem dimer of the monomeric red fluorescent protein CHERRY (2xmCH) (*49*) was used to generate a stable transgenic Arabidopsis line to increase the poor signal obtain in red, in particular, because of the autofluorescence of the plant tissue.

The swapping domain was engineered using a vector containing mCitrine (mCit), cYFPnoSTOP/pDONR221 [previously described by (*11*)], and a vector containing MAP65-3 (*27*). The C-terminal domain 2 of MAP65-3 (MAP65-3^Cter^; EALYGSKPSPSKPLGGKKAPRMSTGGASNRRLSLGAAMHQTPKPNKKADHRHNDGALSNGRRGLDIAGLPSRKQSMNPSEMLQSPLVRKPFSPISTTVVASKANIATTTTQQLPKNNAVNEISSFATPIKNNNILRNLEEEKMMTMMMQTPKNVAAMIPIPSTPATVSVPMHTAPTPFTNNARLMSEKPEVVEYSFEERRLAFMLQSECRLV) was amplified by polymerase chain reaction (*24*): FW: ccaactttgtacaaaaaagcaggctttaaccatggaggcactttacgggtccaaaccc; Rv: gaacagctcctcgcccttgctcaccataaccaaacgacattcagactgtagcatgaa) and introgressed into cYFPnoSTOP/pDONR221 by Gibson cloning (primer FW: ccttcatgctacagtctgaatgtcgtttggtaatggtgagcaagggcgaggagctgt Rv: gctgggtttggacccgtaaagtgcctccatggttaagcctgcttttttgtacaaagtt). Then, by directed mutagenesis, we add an ATG before MAP65-3^Cter^ sequence (primer FW:gcaggcttaaccATGgaggcactttacggg Rv:cccgtaaagtgcctcCATggttaagcctgc). MAP65-3^Cter^ -cYFPnoSTOP/pDONR221vector was used to obtain final destination vectors using three fragments LR recombination system (Thermo Fisher Scientific, www.thermofisher.com) using pB7m34GW destination vector (*48*), pDONR-P4-P1R containing MAP65-3 promotor, and pDONOR-P2R-P3 containing SAC9.

WT Col-0 and heterozygous (or homozygous) *sac9-3* were transformed using the dipping method (*50*). For each construct generated in this paper, between 20 and 24 independent T1 were selected on antibiotics and propagated. In T2, all lines were screened using confocal microscopy for fluorescence signal and localization. Between 3 and 5 independent lines with a mono-insertion and showing a consistent, representative expression level and localization were selected and grown to the next generation. Each selected line was reanalyzed in T3 by confocal microscopy to confirm the results obtained in T2 and to select homozygous plants. At this stage, we selected one representative line for in depth analysis of the localization and crosses and two representative lines for in depth analysis of mutant complementation.

### Live cell imaging

Time-lapse imaging on living root tissues were acquired either manually, or using an automated root tracking system set-up previously (*33*). Briefly, plants roots expressing fluorescent proteins were imaged with a spinning disk confocal microscope while they grow using automatic movement of the microscope stage that compensates for root growth, allowing the imaging of the dividing cells in the root meristem over time (*33*). For both types of experiment, Z-stacks were acquired every 1 to 3 min with the following spinning disk confocal microscope setup: inverted Zeiss microscope (AxioObserver Z1, Carl Zeiss Group, www.zeiss.com/) equipped with a spinning disk module (CSU-W1-T3, Yokogawa, www.yokogawa.com) and a ProEM+ 1024B camera (Princeton Instrument, www.princetoninstruments.com/) or Camera Prime 95B (www.photometrics.com) using a 63× Plan-Apochromat objective (numerical aperture 1.4, oil immersion). GFP and mCit were excited with a 488-nm laser (150 mW), and fluorescence emission was filtered by a 525/50-nm BrightLine! a single-band band-pass filter (Semrock, www.semrock.com/); mCHERRY and TdTOM were excited with a 561-nm laser (80 mW), and fluorescence emission was filtered by a 609/54-nm BrightLine! a single-band band-pass filter (Semrock). For quantitative imaging, pictures of epidermal root meristem cells were taken with detector settings optimized for low background and no pixel saturation. Care was taken to use similar confocal settings when comparing fluorescence intensity or for quantification. In this study, we used PI4P biosensors as a general marker for endomembrane as it localizes both to plasma membrane and trans-Golgi network (*19*). This allows the visualization of both the plasma membrane and the cell plate (*19*), in *sac9-3* and WT plants. We are not expecting a change in behavior for this biosensor in *sac9-3* as we observed the same localization in the WT (*12*). For the double localization of the membranes and the microtubules in sac9-3 using time-lapse imaging, the fact that both markers are tagged with similar fluorophores is not optimal. We were only able to rescue plants for the cross *sac9-3^−/−^* × RFP-MBD. Then we tried to introduce a third construct in *sac9-3^−/−^* × RFP-MBD with a mCit tagged membrane marker, but we did not succeed. Therefore, because of technical limitations, we analyzed the line expressing sac9-3^−/−^ × RFP-MBD × 2xmCh-2xPH^FAPP1^ and the corresponding RFP-MBD × 2xmCherry-2xPH^FAPP1^ in WT.

### Calcofluor staining and immunolocalization imaging

For calcofluor staining, root meristem cell walls were stained using the calcofluor dye, following the protocol described in (*51*). Seedlings were incubated overnight in a fixation buffer (50% of methanol, 10% of acetic acid, and 40% of distilled water). The seedlings were then rehydrated in ethanol baths for 10 min each: 50% ethanol, 30% ethanol, and distilled water twice. Afterward, the seedlings were transferred in the staining solution for overnight incubation {90% of clearsee solution (5% of urea, 15% of deoxycholic acid, and 10% of xylitol in distilled water) and 10% of calcofluor white solution [500 mg of Fluorescent Brightener 28 in distilled water (qsp 50 ml) and 1 drop of NaOH 10 N]}. Before imaging, the seedlings were rinsed for 15 min in clearsee solution. For segmentation and tissue localization of the defects, z-stacks were performed with 0.39-μm space between acquisitions. For *sac9-3* defects comparison with MAP65-3^Cter^ complementation experiments, z-stacks were performed with 0.8 to 1 μm space between acquisitions.

Whole-mount immunolocalization was performed as described (*52*). For immunolocalization, seedlings were fixed in 4% paraformaldehyde and 0.1% Triton X-100 in ½ MTSB buffer [25 mM Pipes, 2.5 mM MgSO_4_, and 2.5 mM EGTA (pH 6.9)] for 1 hour under vacuum and then rinsed in phosphate-buffered saline (PBS) 1X for 10 min. Samples were then permeabilized in ethanol for 10 min and rehydrated in PBS for 10 min. Cell walls were digested using the following buffer for 1 hour: 2 mM MES (pH 5), 0.20% driselase, and 0.15% macerozyme. Tissues were incubated overnight at room temperature with the B-5-1-2 monoclonal anti-α-tubulin (Sigma-Aldrich) and the anti-KNOLLE antibody [gift of G. Jürgens, University of Tübingen, Germany (*44*)]. The next day, tissues were washed for 15 min in PBS and 50 mM glycine, incubated with secondary antibodies (Alexa Fluor 555 goat anti-rabbit for KNOLLE antibody and Alexa Fluor 488 goat anti-mouse for the tubulin antibody) overnight, and washed again in PBS and 50 mM glycine. Samples were incubated in 10% calcofluor white solution for 2 hours and then mounted in VECTASHIELD.

Imaging was performed on an inverted Zeiss CLSM800 confocal microscope using a 40× Plan-apochromatic objective. Dual-color images were acquired by sequential line switching, allowing the separation of channels by both excitation and emission. GFP was excited with a 488-nm laser, mCIT was excited with a 515-nm laser, mCH/tdTOM were excited with a 561-nm laser, and last, Fluorescent Brightener 28 (Calcofluor) was recorded using a 405-nm excitation.

### Super-resolution microscopy

For imaging mCit-SAC9 in fixed tissue, seedlings expressing mCit-SAC9 were fixed in 4% paraformaldehyde and 0.1% Triton X-100 in ½ MTSB buffer [25 mM Pipes, 2.5 mM MgSO_4_, and 2.5 mM EGTA (pH 6.9)] for 1 hour under vacuum and then rinsed in PBS 1X for 10 min. Samples were then permeabilized in methanol for 10 min and rehydrated in PBS for 10 min. Cell walls were digested using the following buffer for 1 hour: 2 mM MES (pH 5), 0.20% driselase, and 0.15% macerozyme. Tissues were incubated overnight at room temperature with the B-5-1-2 monoclonal anti-α-tubulin (Sigma-Aldrich). The next day, tissues were washed for 15 min in PBS and 50 mM glycine, incubated with secondary antibodies (Alexa Fluor 555 against the B-5-1-2 monoclonal anti-α-tubulin and with the GFP-Booster Atto647 antibody) overnight, and washed again in PBS and 50 mM glycine. Samples were incubated in 10% calcofluor white solution for 50 min and then mounted in 80% Citifluor AF1.

High-resolution imaging was performed using a LSM980 Airyscan2 (ZEISS) using a 40× objective (numerical aperture 1.3, oil immersion). Dual-color images were acquired by sequential line switching, allowing the separation of channels by both excitation and emission. GFP-Booster, mCitrine, mTU2, and Fluorescent Brightener 28 (Calcofluor) were excited using 639-, 514-, 445-, and 405-nm lasers, respectively. All the signals were recorded with an airyscan 2 detector except Fluorescent Brightener 28 for which a photomultiplier tube detector was used. Airyscan processing was performed with ZEN imaging software (ZEN blue).

### Root growth quantification

Plants growth was manually measured using ImageJ at 7 days post-germination. For each drug, three independent biological replicates were analyzed (two for latrunculin B) with each time three technical replicates.

### Segmentation and tissue localization of *sac9-3* defects

Quantification of cell division defects was based on manual counting of full Z-stacks with a z-spacing equal to the lateral resolution to get cubic voxels allowing to quantify of the defects, number, and position in the tissue, with the ImageJ orthogonal view plugin (or z-spacing of 0.5 and 1 μm in cases that only defects count was needed). Segmentation was performed using ImageJ (morphological segmentation) plugin as described and angle extraction using ImageJ plugin developed (*53*). The *sac9-3* defects architecture was manually measured using ImageJ, “straight line” and “angle tool” tools, and data were analyzed with Excel.

### Fluorescence intensity extraction and localization indexes (fig. S21)

Normalized fluorescence intensity: This was obtained using the Fiji tool, extracted from 10 dividing cells [cells of an approximatively equivalent length, normalized to the exact same size by suppressing a part of the space between the cell plate and plasma membrane (Disk phragmoplast) or just the exact same length on only one expanding edge (discontinuous phragmoplast), normalized by the intensity at the cell plate center (except for figs. S4 and S5 where the normalization was by the cytosolic intensity)], and plotted to obtain an average intensity profile.

For PI(4,5)P_2_ localization index on the discontinuous phragmoplast: Phragmoplast length was measured and divided into two equal parts, the leading zone (domain close to the plasma membrane) and the lagging zone (domain close to the cell center). Fluorescence intensity was measured on each zone of the phragmoplast and, in addition, on the entire cell plate and cytosol. Next, the cytosol intensity background was subtracted and, last, the leading and lagging zones were divided by the global cell plate intensity. The final ratio superior to 1 underlined an enrichment at a specific zone of the phragmoplast. The final ratio inferior to 1 underlines the depletion.

SAC9-PI(4,5)P_2_ localization index on the discontinuous phragmoplast: To address mCit-SAC9 and tdTOM-SAC9^C459A^ co-exclusion with PI(4,5)P_2_ biosensor we used the same index as the PI(4,5)P_2_ localization index. Because we showed that the PI(4,5)P_2_ biosensor was excluded from the leading zone, when a microtubule marker was not present in the imaged transgenic line, we decided to use the PI(4,5)P_2_ biosensor as a separation between the leading and lagging zone. Using PI(4,5)P_2_ biosensor fluorescence decrease as a marker of this separation, we measured the intensity on 1.5 μm (the approximative half-length of the phragmoplast) on each side of PI(4,5)P_2_ biosensor limit. Last, the leading and lagging zones were divided by the intensity of the cell plate and nearby cytoplasm (1 μm around the cell plate).

MAP65-3 labeling distance: Measurement was performed using ImageJ, a “straight line” tool every 2 min on one side of the cell plate (the one with the persistent pool in *sac9-3*). Lines were created between the outer and inner MAP65-3 on the entire length labeled. The lengths were plotted relative to the “disk”-"ring” phragmoplast transition (0 min) and cell plate attachment (14 min).

PI(4,5)P_2_ phragmoplast accumulation index: One dividing cell and one of its surrounding nondividing cells were taken for each root. For every two cells, the signal intensity was measured in one elliptical region of interest (ROI) at the phragmoplast (around the cell plate across all cell lengths or at the equator of interphasic cells), with an equal area between the two cells. In addition, we measured signal intensity at the plasma membrane separating the two cells. Ratios were obtained by dividing both elliptical regions by the signal intensity at the plasma membrane (obtaining the “dividing ratio” for the dividing cell and the “nondividing ratio” for the nondividing cell). Last, the ratio between the “dividing ratio” and the “nondividing ratio” was analyzed. Final ratios equal to 1 indicated that there was no enrichment during cell division. Final ratio greater than 1 indicated an enrichment during cell division at the phragmoplast. Final ratio less than 1 indicated a depletion during cell division at the phragmoplast. This index was performed on the young dividing cells where the cell plate is expanding and not attached and in old dividing cells where the cell plate is partially attached to the plasma membrane (discontinuous phragmoplast).

PI4P accumulation index on the cell plate: The intensity of 2xmCh-2xPH^FAPP1^ signals was quantified on the cell plate at the final cell plate expansion (late ring or discontinuous phragmoplast). Two ROIs were designed, one on the entire cell plate, and the other on the apical plasma membrane. The measured intensity at the cell plate was then normalized (divided) by the measured intensity at the plasma membrane of the same cell as described in (*18*).

KNOLLE accumulation index on the cell plate: The intensity of YFP-KNOLLE signals was quantified on the cell plate at the final cell plate expansion (late ring or discontinuous phragmoplast). Two ROI were designed, one on the entire cell plate, and the other on the cytoplasm. The measured intensity at the cell plate was then normalized (divided) by the measured intensity on the cytoplasm of the same cell as described in (*18*).

Phragmoplast measurements: Phragmoplast width and length were manually measured using ImageJ, “straight line” tool as represented in fig. S10B.

### Statistical analysis

We performed all our statistical analyses in R (version 3.5.0 (2018-04-23), using R studio interface and the packages ggplot2 (*54*), lme4 (*55*), car (*56*), multcomp (*57*), and means (*58*). Graphs were obtained with R and R-studio software and customized with Inkscape (https://inkscape.org). The number of division defects was compared using a generalized linear model with a Poisson law (when effective>20 or using a Quasi-Poisson law if under 20).

Fluorescence intensity indexes were compared using an analysis of variance (ANOVA) statistical test and a Tukey HSD post hoc analysis if the normality was verified or using a Kruskal-Wallis test, Dunn (Bonferroni method) post hoc analyses, if not. All the statistics are found in tables S2 to S14.

## Notes

View/request a protocol for this paper from *Bio-protocol*.
