# Supplementary material for: The phosphoinositide signature guides the final step of plant cytokinesis
Source: Sci Adv. 2023 Jul 19;9(29):eadf7532. doi: 10.1126/sciadv.adf7532 (PMC10355833; doi:10.1126/sciadv.adf7532)
Supplement: Supplementary file 1 — Figs. S1 to S21 Legends for tables S1 to S14 Legends for movies S1 to S10 [file sciadv.adf7532_sm.pdf]

Science Advances
AAAS

# Supplementary Materials for

## The phosphoinositide signature guides the final step of plant cytokinesis

Alexis Lebecq *et al.*

Corresponding author: Marie-Cécile Caillaud, marie-cecile.caillaud@ens-lyon.fr

*Sci. Adv.* **9**, eadf7532 (2023)
DOI: 10.1126/sciadv.adf7532

**The PDF file includes:**

Figs. S1 to S21
Legends for tables S1 to S14
Legends for movies S1 to S10

**Other Supplementary Material for this manuscript includes the following:**

Tables S1 to S14
Movies S1 to S10

# SUPPLEMENTARY FIGURES

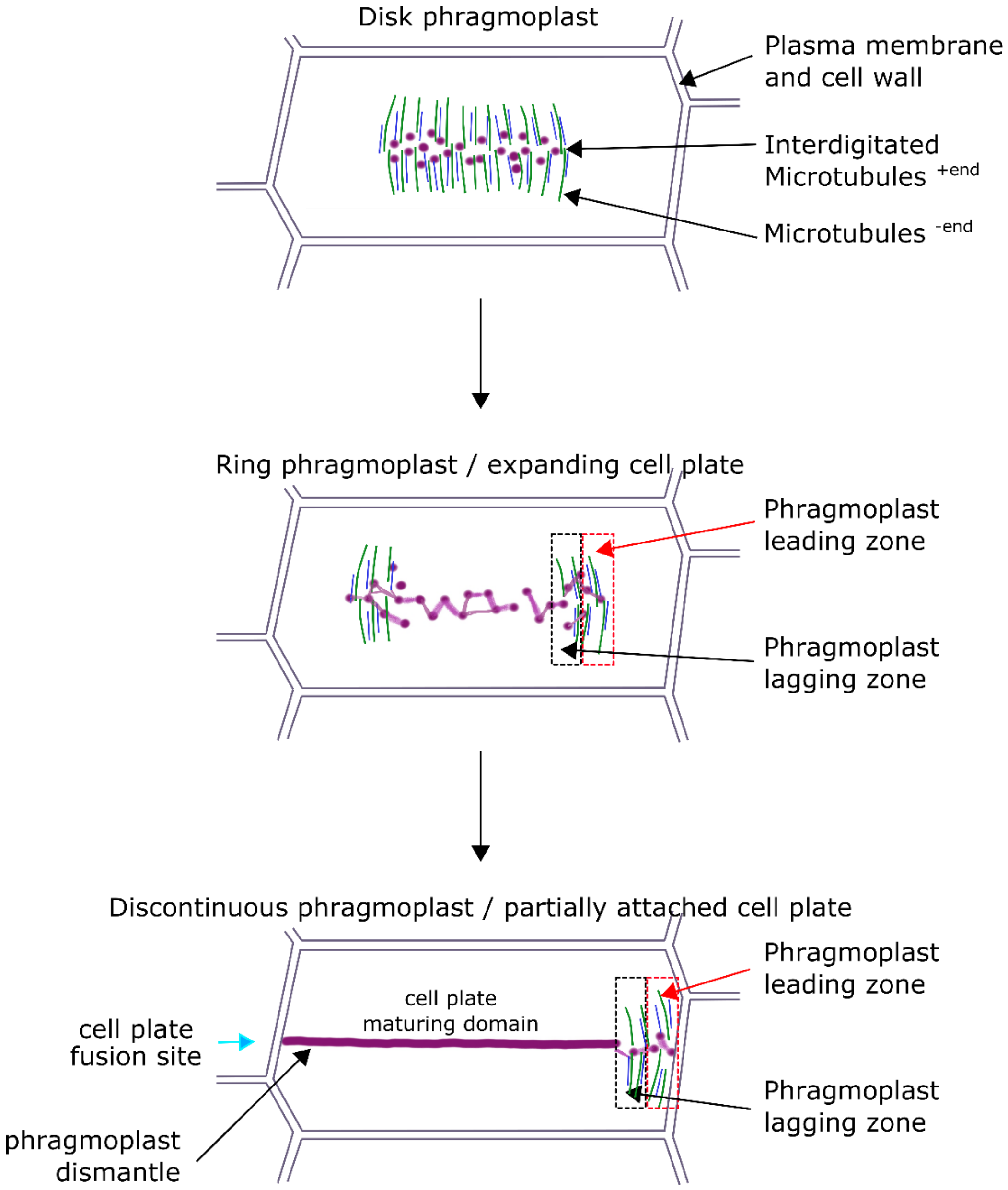


**Fig. S1 | Cell cytokinesis from a cytoskeleton point of view.**

Schematic representation of phragmoplast expansion in a 2D cell. The three main phragmoplast structures are presented: form a Disk phragmoplast until a Discontinuous phragmoplast morphology. Key cytoskeleton's structures are presented: Microtubules plus ends, minus ends, phragmoplast leading and lagging zones and cell plate maturing domain. Microtubules, green; Actin, blue, Cell plate, magenta.

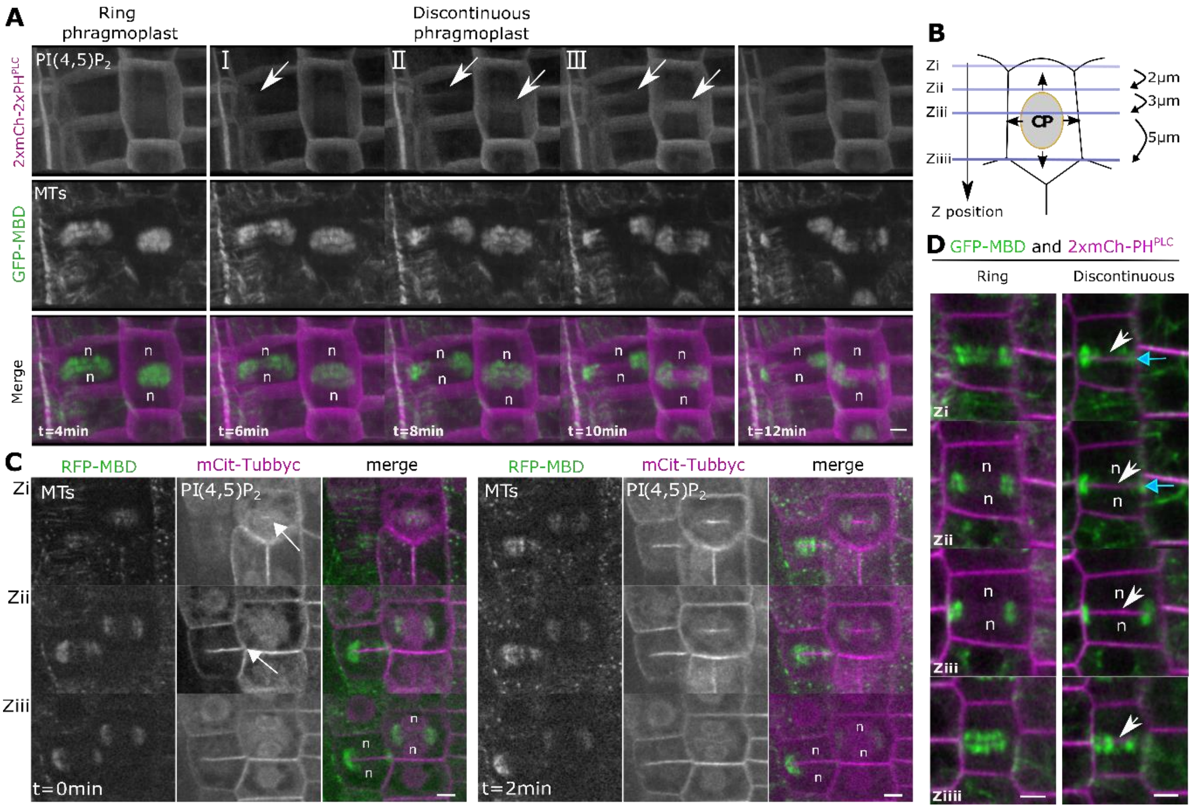


## Fig. S2 | PI(4,5)$P_2$ appearance at the maturing cell plate in wild-type plants

**A**, Z-projection of dividing cells expressing 2xmCh-2xPH$^{PLC}$ together with GFP-MBD. The image was taken every 2 min and 15 optical sections were used for Z-projection. I, II and III images correspond to the sequence of events from the first appearance of PI(4,5)$P_2$ biosensor at the cell plate. **B**, Representation of the different focal planes used in this study from the subcortical part of the cells (Zi, top panel) to the bottom cells (Ziiii, bottom panel). CP, cell plate **C**, Confocal images of WT at different Z, expressing *mCit-Tubbyc* together with RFP-*MBD* during the discontinuous phragmoplast stage, in wild type plants. **D**, Confocal images of WT, expressing *2xmCh-2xPH$^{PLC}$* together with *GFP-MBD* during the discontinuous phragmoplast stage, from the subcortical part of the cells (Zi, top panel) to the center of the cells (Ziii, bottom panel) in WT. White arrow, PI(4,5)$P_2$ biosensor appearance; blue arrow, cell plate fusion site; Black arrow, cell plate direction of growth; n, nucleus; scale bars, 5µm.

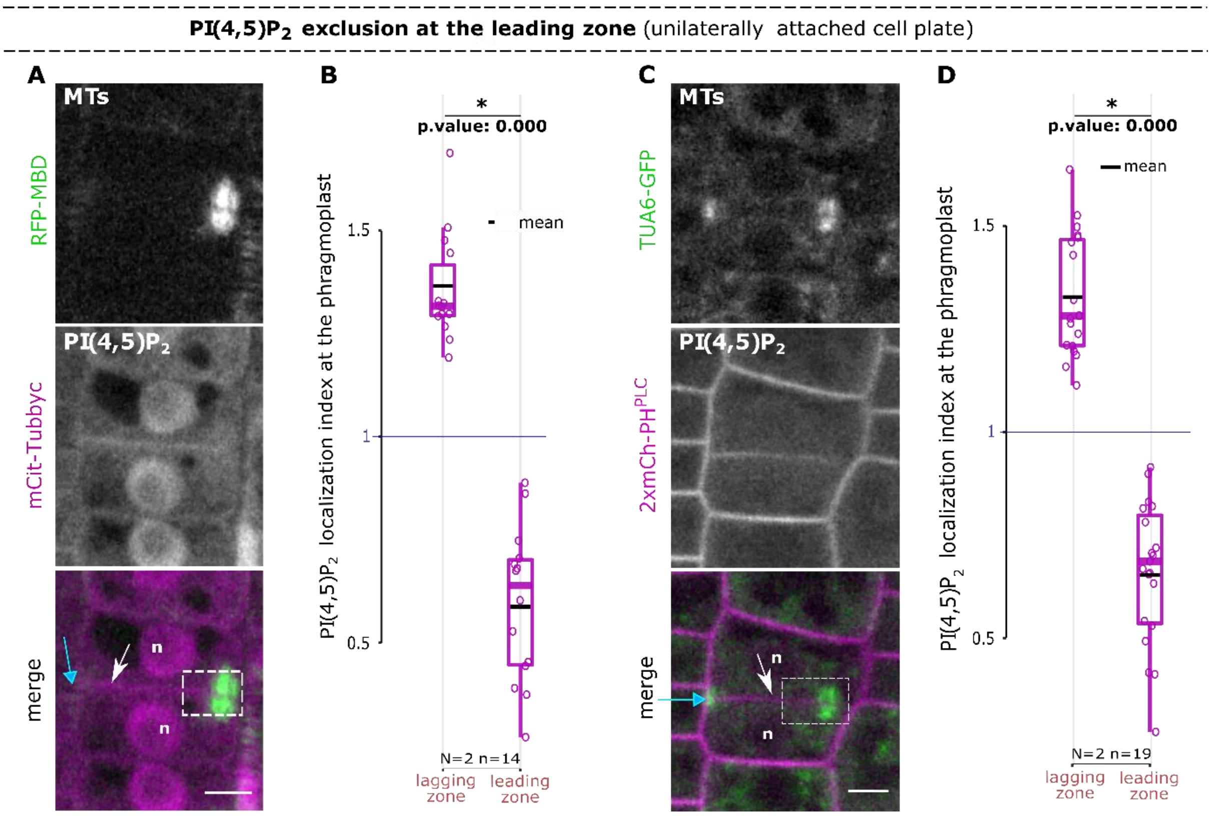


## Fig. S3 | PI(4,5)$P_2$ is absent from the cell plate leading zone in WT

**A, C,** Confocal images of WT, expressing respectively *mCit-Tubbyc* with *RFP-MBD* (A) or *2xmCh-2xPH*$^{PLC}$ with *TUA6-GFP* (C) at the discontinuous phragmoplast. **B, D**, Quantification of PI(4,5)$P_2$ localization index for PI(4,5)$P_2$ biosensors (mCit-Tubbyc and 2xmCh-2xPH$^{PLC}$) with respect to two microtubules reporter (RFP-MBD or TUA6-GFP respectively), normalized by cell plate intensity. “1” blue line represents a situation in which there is no biosensor enrichment. In the plots, middle horizontal bars represent the median, while the bottom and top of each box represent the 25$^{th}$ and 75$^{th}$ percentiles, respectively. At most, the whiskers extend to 1.5 times the interquartile range, excluding data beyond. For range of value under 1,5 IQR, whiskers represent the range of maximum and minimum values. Results of the statistical analysis (shown in the supplementary table) are presented (N = number of replicates, n = number of cells). White arrow, PI(4,5)$P_2$ biosensor appearance; Blue arrow, cell plate fusion site; dotted lines, region of interest; n, nucleus; scale bars, 5µm.

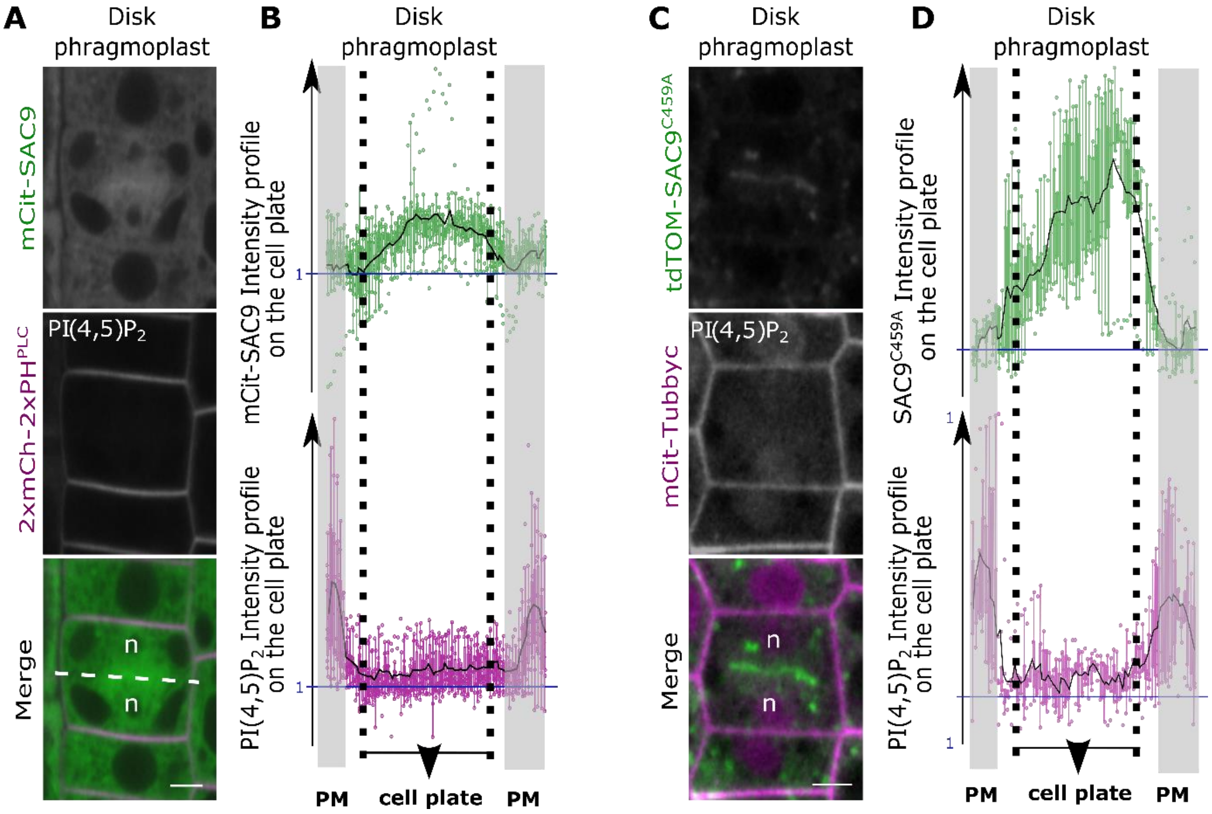


**<u>Fig. S4 | SAC9 and the PI(4,5)$P_2$ localization during the disk phragmoplast stage</u>**

**A**, Confocal images of mCit-SAC9 and 2xmCh-2xPH$^{PLC}$ at the "Disk phragmoplast" phase in WT. **B**, Normalized intensity profiles of mCit-SAC9 (top panel) and 2xmCh-2xPH$^{PLC}$ (bottom panel) during the Disk phragmoplast phase (entire cell plate, dotted line). **C**, Confocal images of WT expressing *tdTOM-SAC9$^{C459A}$* and *mCit-Tubbyc*. **D**, Normalized intensity profiles of tdTOM-SAC9$^{C459A}$ (top panel) and mCit-Tubbyc (bottom panel).

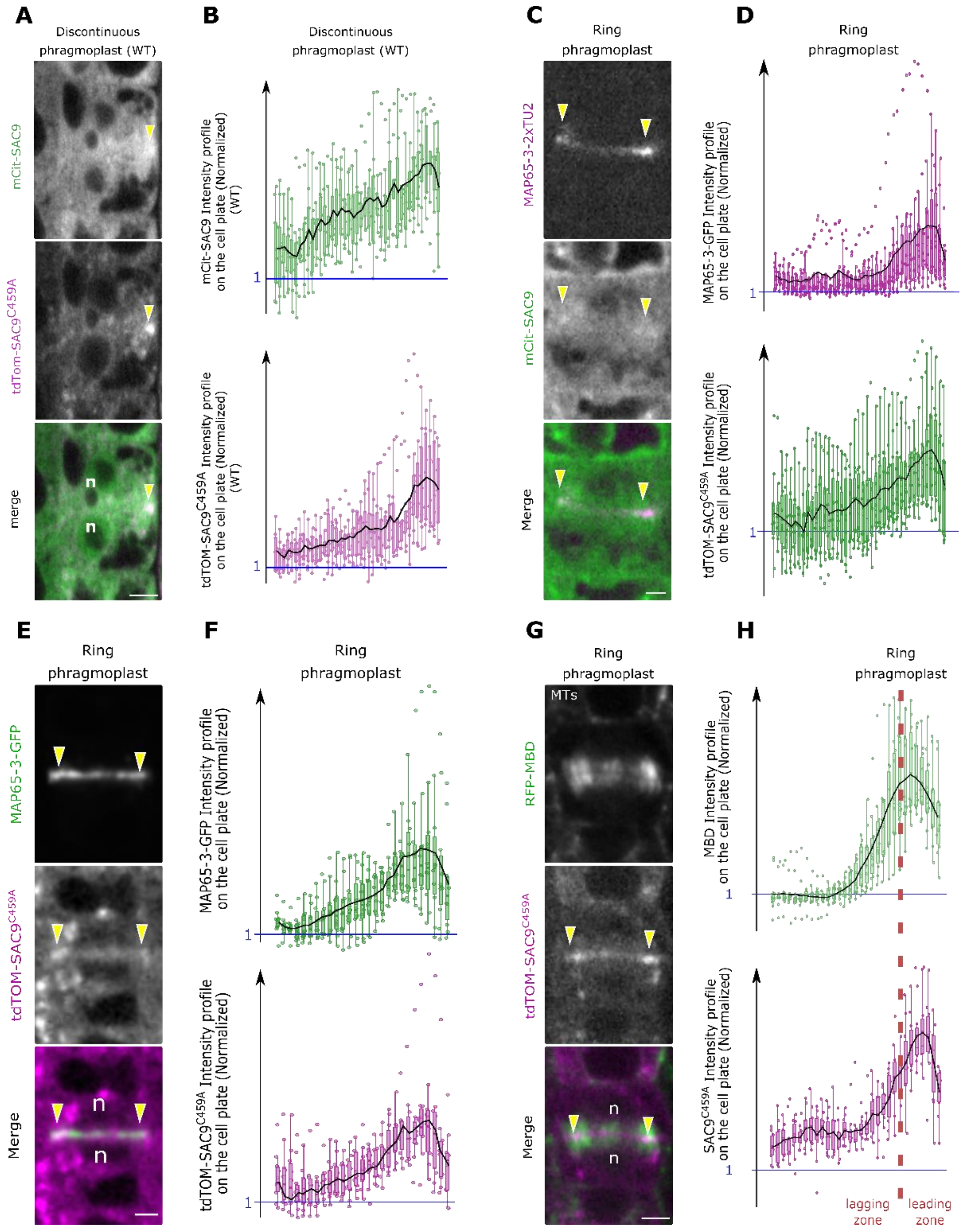


**<u>Fig. S5 | SAC9 colocalizes with SAC9$^{C459A}$ and MAP65-3 at the phragmoplast leading zone during cytokinesis</u>**

**A**, Images of mCit-SAC9 with MAP65-3-2xmTU2. **B**, Normalized intensity profiles corresponding to (A). **C**, Images of tdTOM-SAC9$^{C459A}$ with MAP65-3-GFP. **D**, Normalized intensity profiles corresponding to (C). **E**, Images of tdTOM-SAC9$^{C459A}$ and GFP-MBD at the ring phragmoplast stage. **F**, Normalized intensity profiles corresponding to (E). **G**, Images of mCit-SAC9 with tdTOM-SAC9$^{C459A}$ at the discontinuous phragmoplast stage. **H**, Normalized intensity profiles corresponding to (G). Yellow arrowhead, SAC9 and MAP65-3 enrichments at the leading zone; n, nucleus; Scale bar, 5 μm.

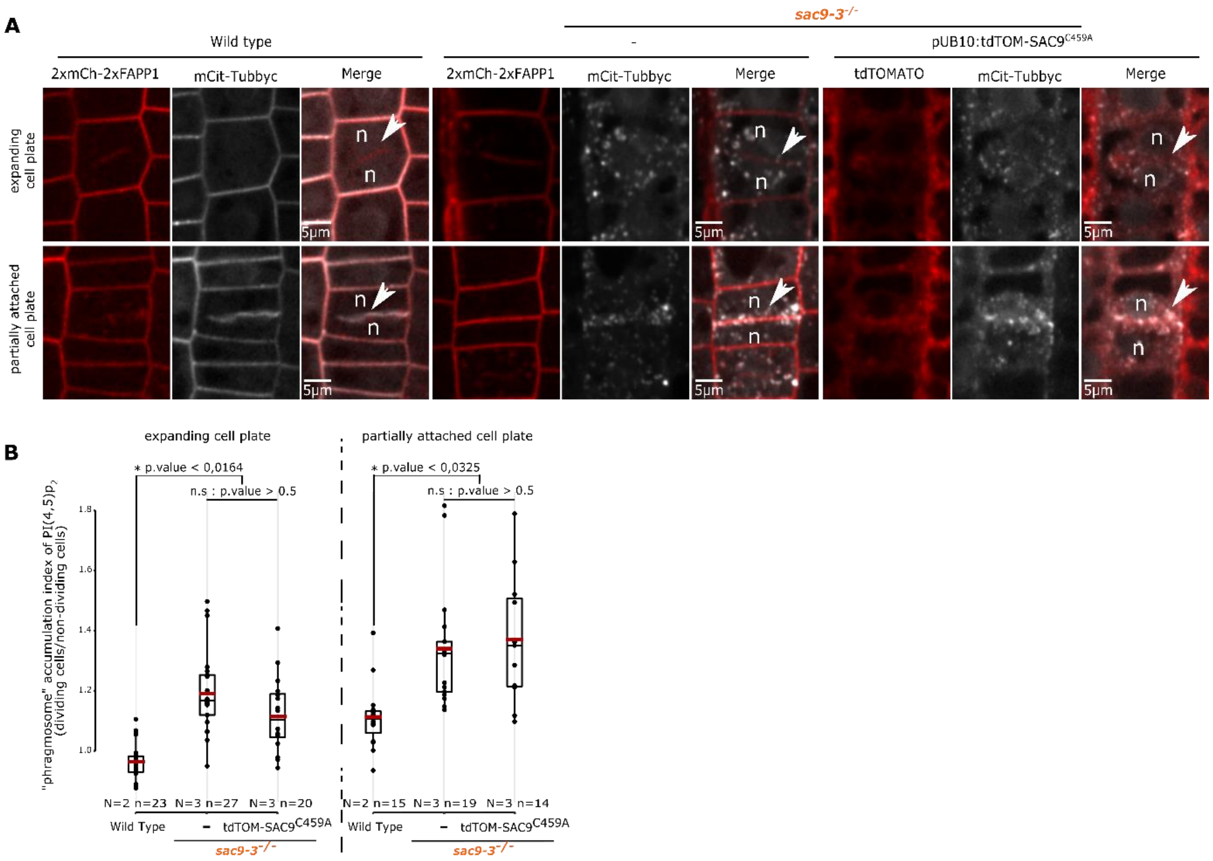


## Fig. S6 | tdTOM-SAC9$^{C459A}$ localization regarding the PI(4,5)$P_2$ biosensor during the discontinuous phragmoplast expansion in WT and *sac9-3*

**A**, Images of mCit-Tubbyc with tdTOM-SAC9$^{C459A}$ in WT. **B**, Images of mCit-Tubbyc with tdTOM-SAC9$^{C459A}$ in *sac9-3*. **C**, Normalized intensity profiles corresponding to (B) during the discontinuous phragmoplast phase. White arrow, PI(4,5)$P_2$ biosensor appearance; White arrowhead, abnormal PI4,5)$P_2$ enrichment at the leading zone in *sac9-3*; Yellow arrowhead, SAC9$^{C459A}$ enrichment at the leading zone; White dotted line, cell contour; Blue arrow, cell plate fusion site; n, nucleus; Scale bars, 5μm.

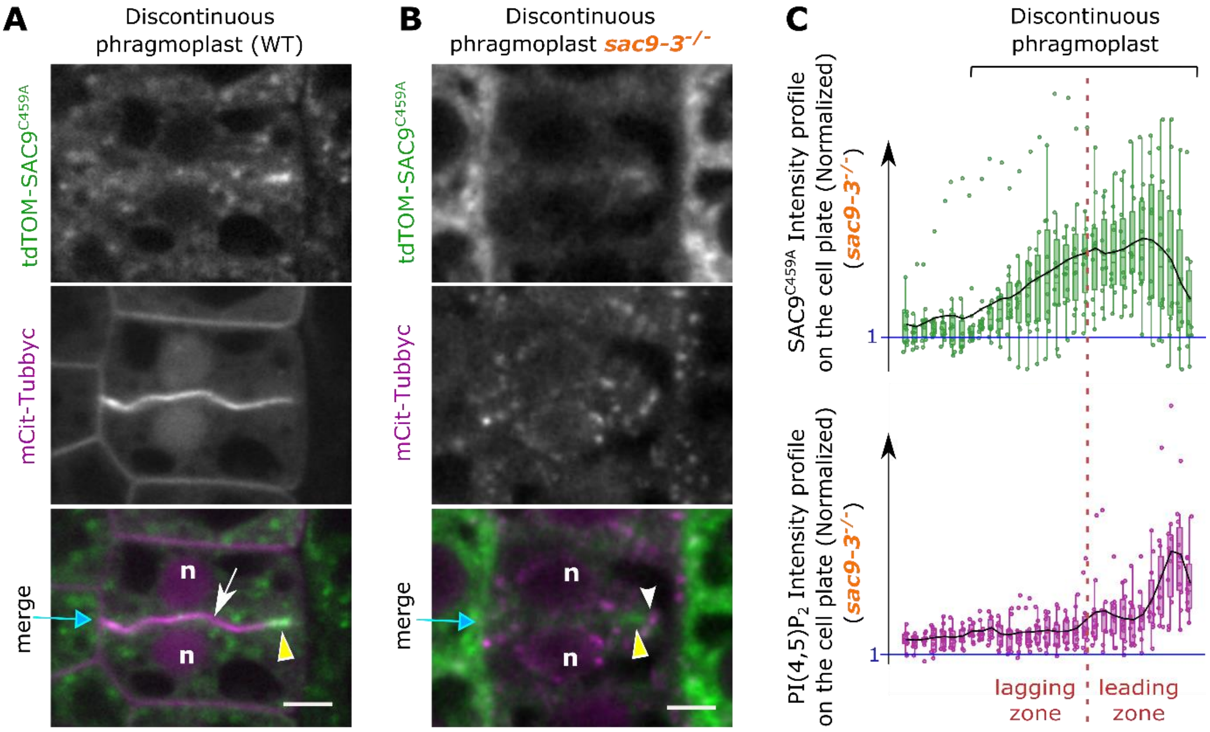


**Fig. S7 | Mutation in the putative catalytic domain of SAC9 does not rescue the PI(4,5)$P_2$ biosensor misregulation during cell division.**

**A**, Confocal images of WT expressing *2xmCh-PH$^{FAPP1}$ (red)*, *sac9-3* expressing *2xmCh-PH$^{FAPP1}$ (red)* and *sac9-3* expressing *tdTOM-SAC9$^{C459A}$*, together with *mCit-Tubbyc* (white). For the sets of three panels, the top images correspond to the "expanding cell plate" phase and the bottom image to the "partially attached cell plate" phase. Here, 2xmCh-2xPH$^{FAPP1}$ is used as a marker for membranes (plasma membrane and cell plate). The white arrow points out the cell plate for PI(4,5)$P_2$, yellow arrowhead the SAC9$^{C459A}$ enrichment and "n" the nucleus (scale bars, 5µm). **B**, Comparison of the phragmosome accumulation index for PI(4,5)$P_2$ biosensors (mCit-Tubbyc) at the "expanding cell plate" (left panel) and partially attached cell plate" (right panel) between WT, *sac9-3* mutant and *sac9-3* expressing *tdTOM-SAC9$^{C459A}$*. In the plots, middle horizontal bars represent the median, while the bottom and top of each box represent the 25$^{th}$ and 75$^{th}$ percentiles, respectively. At most, the whiskers extend to 1.5 times the interquartile range, excluding data beyond. For range of value under 1,5 IQR, whiskers represent the range of maximum and minimum values. Results of the statistical analysis (shown in the supplementary table) are presented (N = number of replicates, n = number of cells).

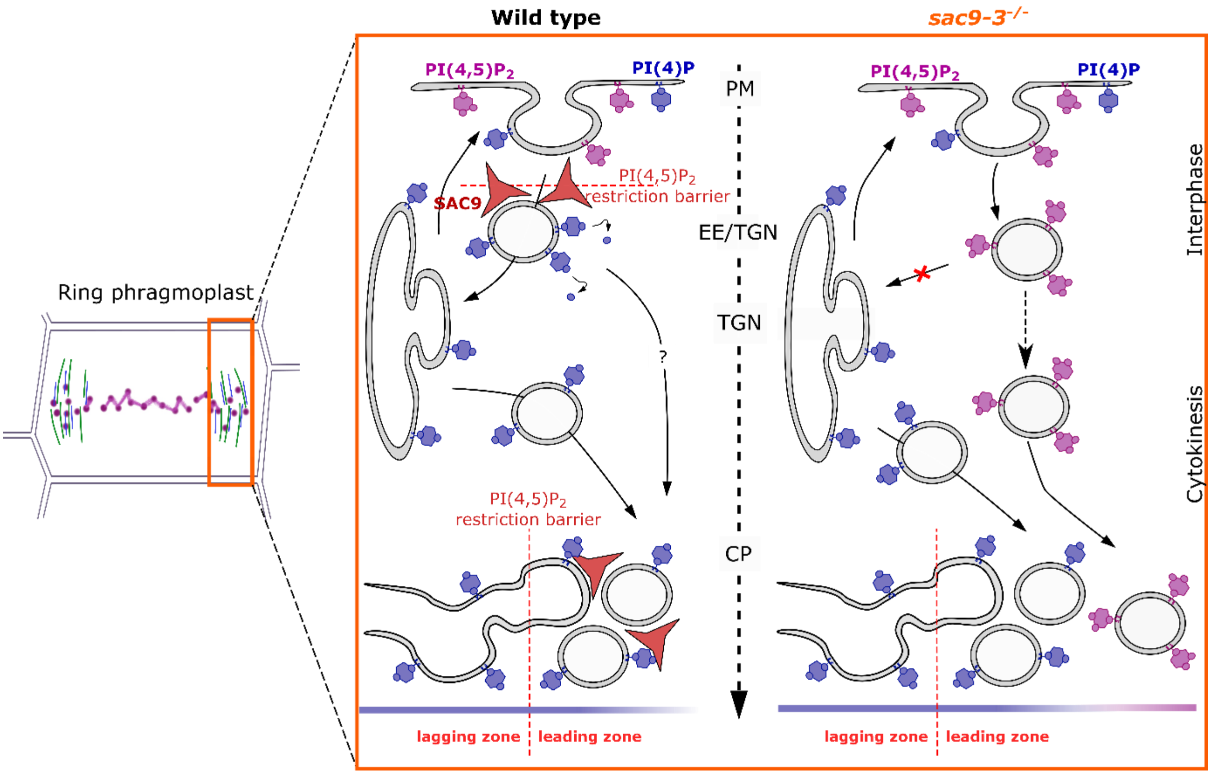


**Fig. S8 | Model for the abnormal recruitment of PI(4,5)$P_2$ in *sac9-3* interphasic cells and mitotic cells**

Hypothetical model of the mechanism at the cell plate edges based on our results. In wild-type plants, SAC9 restricts the PI(4,5)$P_2$ at the plasma membrane giving rise to a endomembrane system free of this lipid. Upon SAC9 absence, the PI(4,5)$P_2$ is accumulated in endomembrane compartments lost in the endocytic pathway, no longer able to reach and fuse to the TGN. This abnormal PI(4,5)$P_2$ containing membranes accumulating in absence of SAC9 are during the cytokinetic process directed at the leading edges of the phragmoplast where they particularly accumulated during its fusion to the plasma membrane.

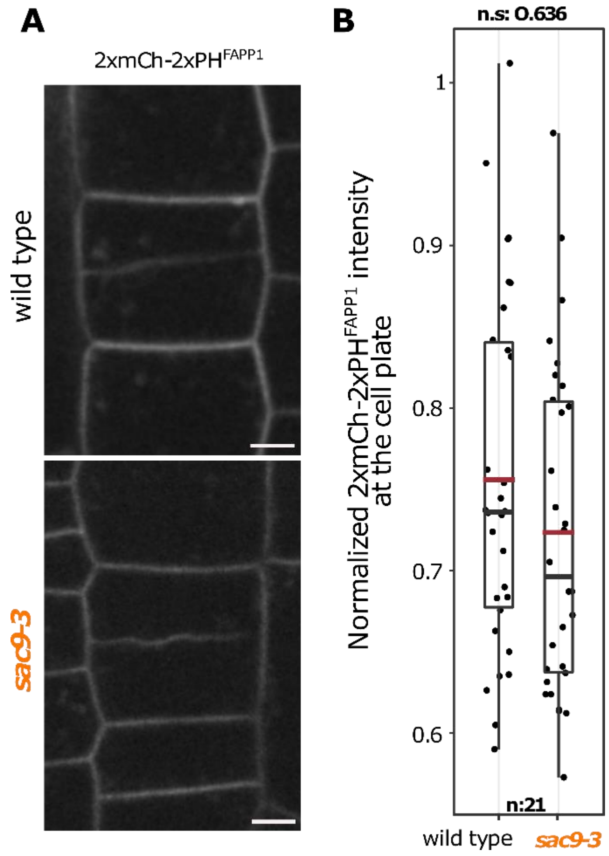


**Fig. S9 | PI4P level at the cell plate is not significantly impacted in *sac9-3*.**

**A**, Confocal images of 2xmCh-2xPH$^{FAPP1}$, PI4P reporter, during the final cell plate expansion (ring and discontinuous phragmoplast) in wild type (upper panel) and *sac9-3* (bottom panel). **B**, comparison of 2xmCh-2xPH$^{FAPP1}$ intensity at the cell plate, normalized by its intensity on the plasma membrane.

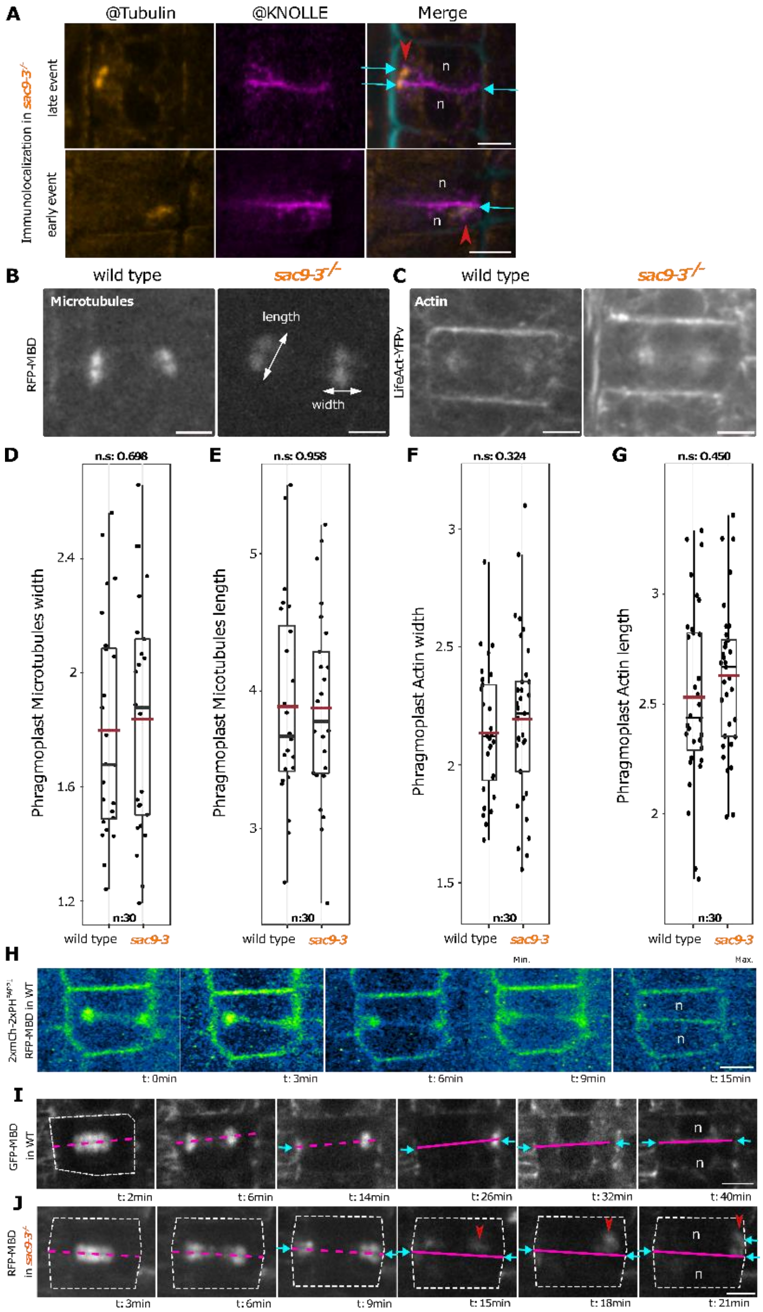


**Fig. S10 | In *sac9-3*, cell plate branching correlates with microtubule perturbation.**

**A**, Co-immunolocalization against αtubulin and KNOLLE in *sac9-3* stained with calcofluor during two steps of *sac9-3*'s defect emergence. Scale bar, 5 µm. **B**, Confocal images of microtubules, RFP-MBD in wild type (left) and *sac9-3* during the ring phragmoplast stage. **C**, Confocal images of actin filament, LifeAct-YFPv in wild type (left) and *sac9-3* during the ring phragmoplast stage. **D**, comparison of microtubules phragmoplast width. **E**, comparison of phragmoplast microtubules length. **F**, Comparison of Actin phragmoplast width. **G**, Comparison of Actin phragmoplast length **H**, Z projection of 2xmCh-2xPH$^{FAPP1}$ and RFP-MBD in WT. Here, the signal is color coded in green fire blue (see scale bar on the right). The signal corresponding to microtubules and the one corresponding to membrane are not distinguished). Note that here, 2xmCh-2xPH$^{FAPP1}$ is used at the membrane marker (plasma membrane and cell plate). **I**, Time-lapse of a Z projection of RFP-MBD in wild type. Scale bar, 5 µm. **J**, Time-lapse of a Z projection of RFP-MBD in *sac9-3*. Scale bar, 5 µm. Blue arrow, cell plate fusion site; White arrow, phragmoplast length and width; Red arrowhead, branched cell plate; n, nucleus.

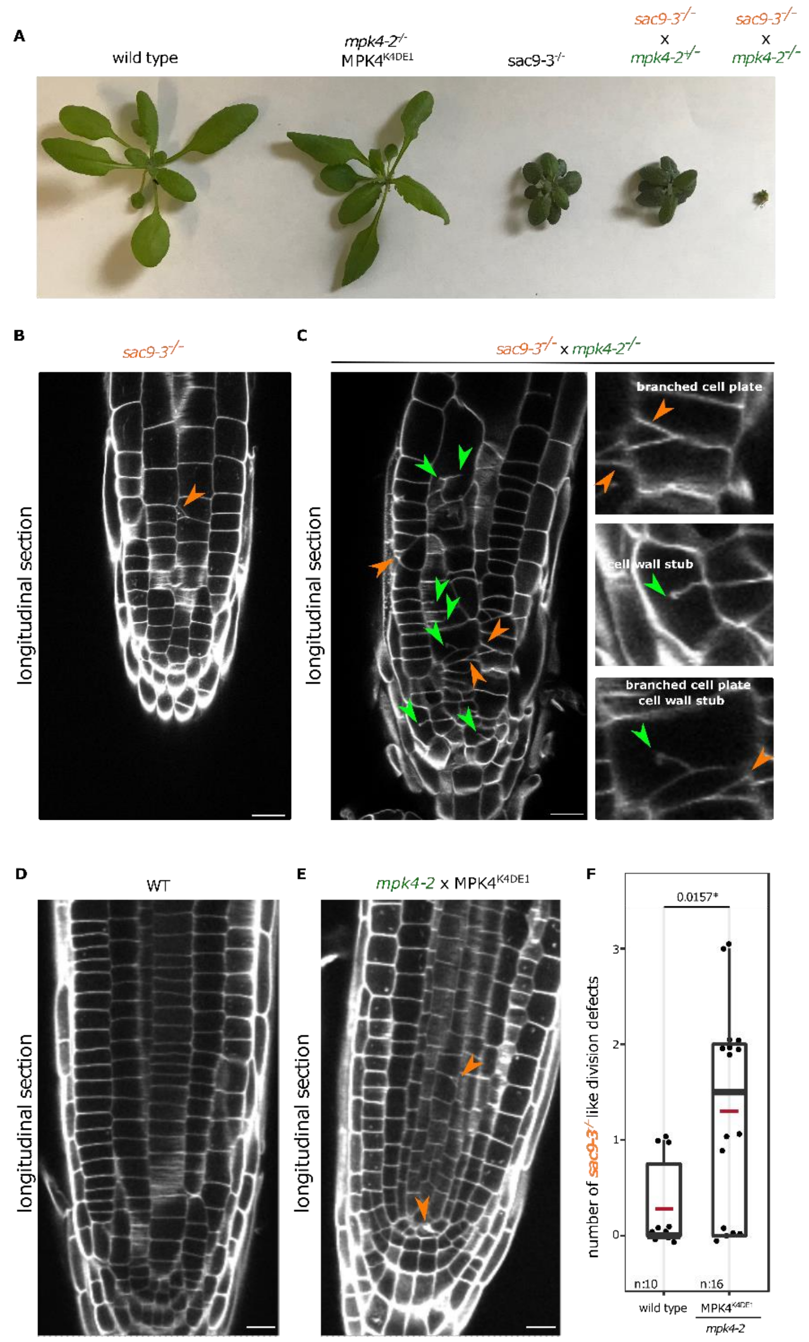


**Fig. S11 | MPK4 activity could be part of *sac9-3* branching defect.**

**A** and **B**, Representative image extracted from a Z-stack of calcofluor-stained fixed roots (21 day-olds) for *sac9-3* (a) and the double mutant *sac9-3 x mpk4-2* (B, left part: crop on defectives cells). **C** and **D**, Representative image extracted from a Z-stack of calcofluor-stained fixed roots (7 day-olds) for wild type (c) and *mpk4-2* complemented with a constitutively active MPK4 (MPK4$^{K4DE1}$). **E**, Quantification of the number of *sac9-3* like defects. The result of the statistical analysis (shown in the supplementary table) is presented. In the plots, middle horizontal bars represent the median, while the bottom and top of each box represent the 25$^{th}$ and 75$^{th}$ percentiles, respectively. At most, the whiskers extend to 1.5 times the interquartile range, excluding data beyond. For range of value under 1,5 IQR, whiskers represent the range of maximum and minimum values. Results of the statistical analysis (shown in the supplementary table) are presented (n = number of cells). Orange arrow, *sac9-3* like cell wall defects; Green arrow: cell wall stubs. Scale bar: 20 µM.

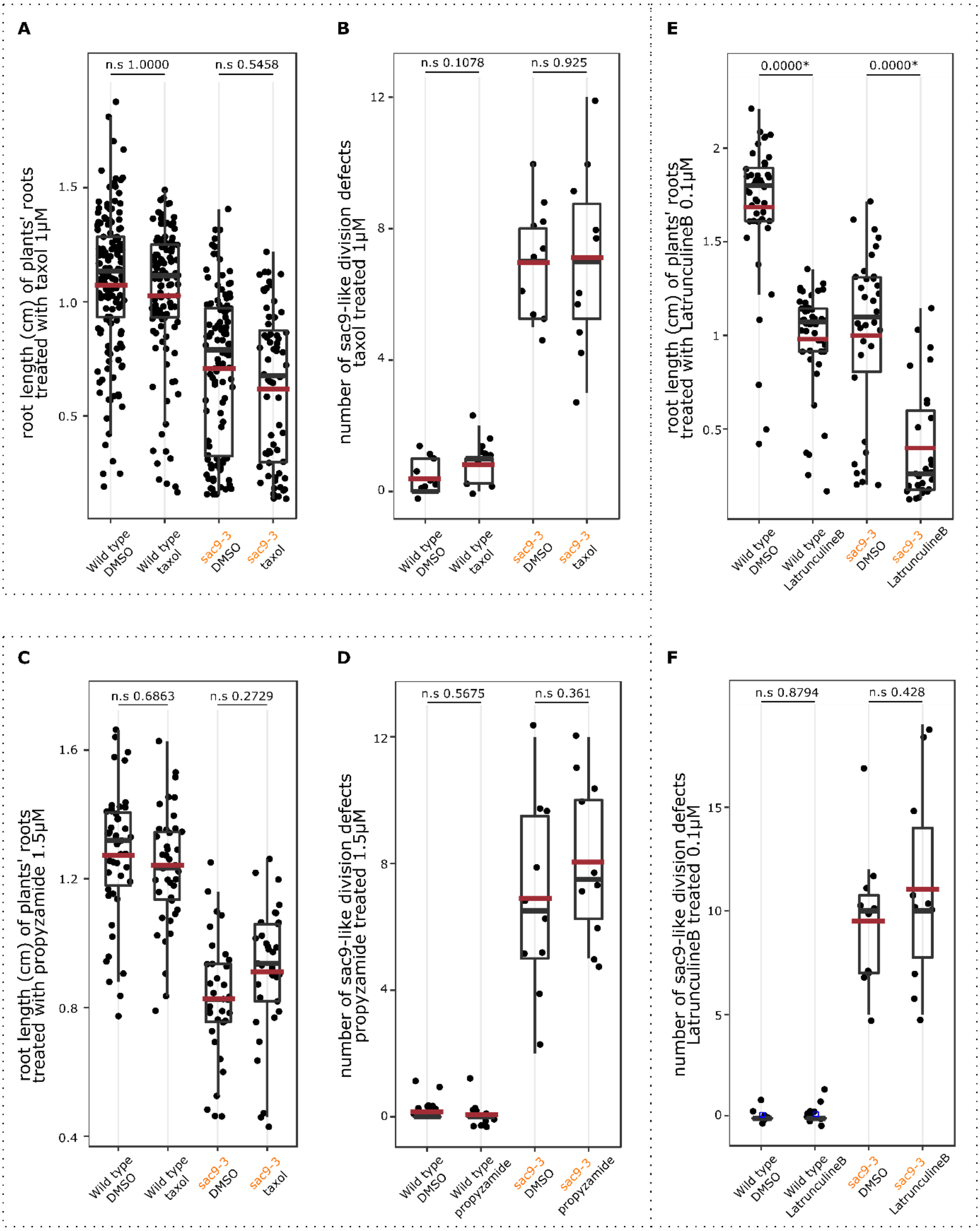


**Fig. S12 | *sac9-3* mutant sensibility to mild cytoskeleton perturbation.**

**A**, **B** Quantification at 7 days post germination (dpg) of WT and *sac9-3* root length grown on ½ MS plates supplemented with 1 μM Taxol (A) or 1.5 μM Propyzamide (B). **C**, **D** Quantification of *sac9-3* like division defects in 7 dpg WT and *sac9-3* plants grown on ½ MS plates supplemented with 1 μM Taxol (C) or 1.5 μM Propyzamide (D). **E** Quantification at 7 days post germination (dpg) of WT and *sac9-3* root length grown on ½ MS plates supplemented with 0.1 μM Latrunculin B. **F**, Quantification of *sac9-3* like division defects in 7 dpg WT and *sac9-3* plants grown on ½ MS plates supplemented 0.1 μM Latrunculin B.

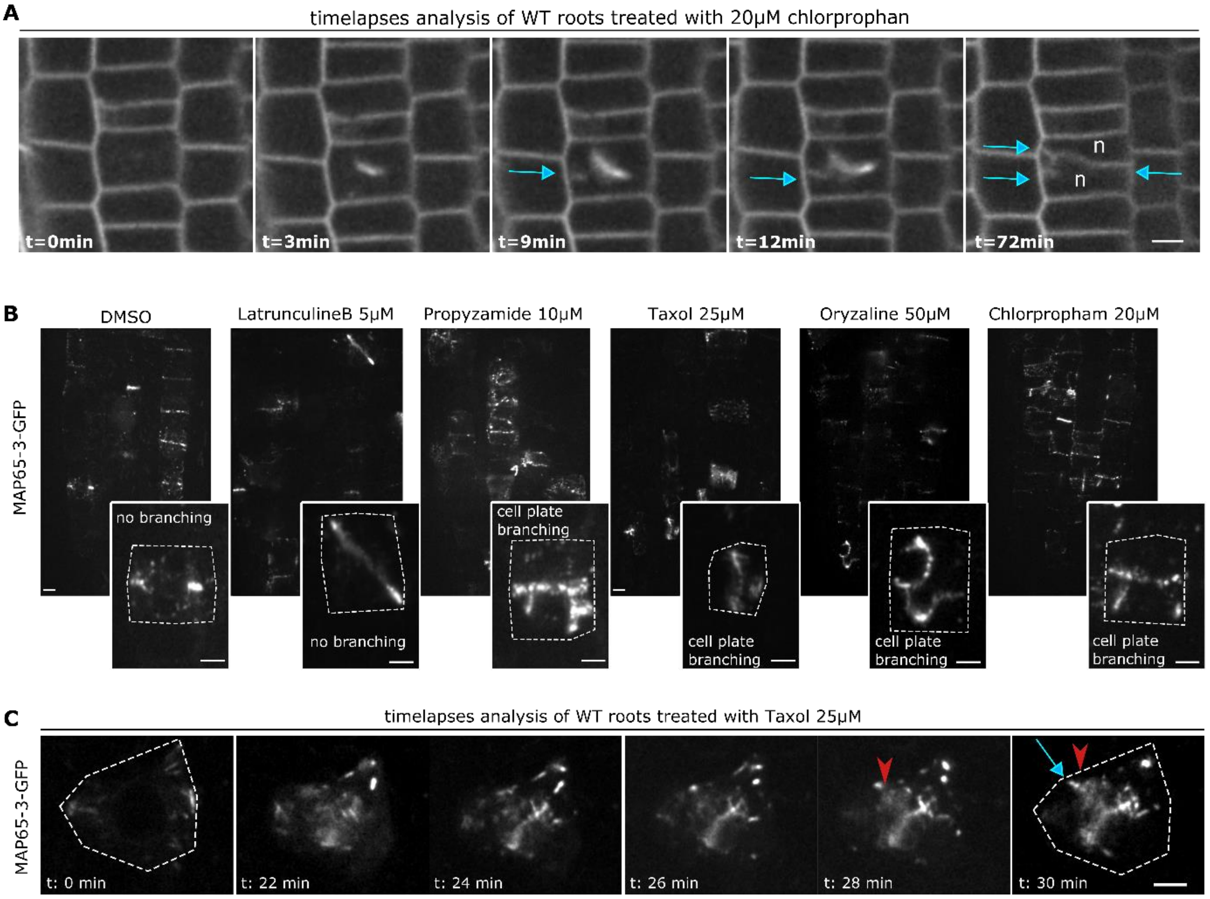


**<u>Fig. S13 | Pharmacological perturbations of microtubules but not actin lead to cell plate branching, yet different from *sac9-3*.</u>**

**A**, image series acquired with a root tracking system of a wild type plant treated for 2 h with chlorpropham 20 µM. **B**, confocal images of wild type plants expressing *MAP65-3-GFP*, treated with (from left to right) 5µm Latrunculin B, 25µM Taxol, 10µM Propyzamide, 50µM Oryzalin and 20µM Chlorpropham respectively. **C**, Image series acquired with a root tracking system of a wild type plant expressing *MAP65-3-GFP* treated for 24 h with Taxol 25 µM. Scale bar, 5 µm. Blue arrow, cell plate fusion site; Red arrowhead, branched cell plate; n, nucleus.

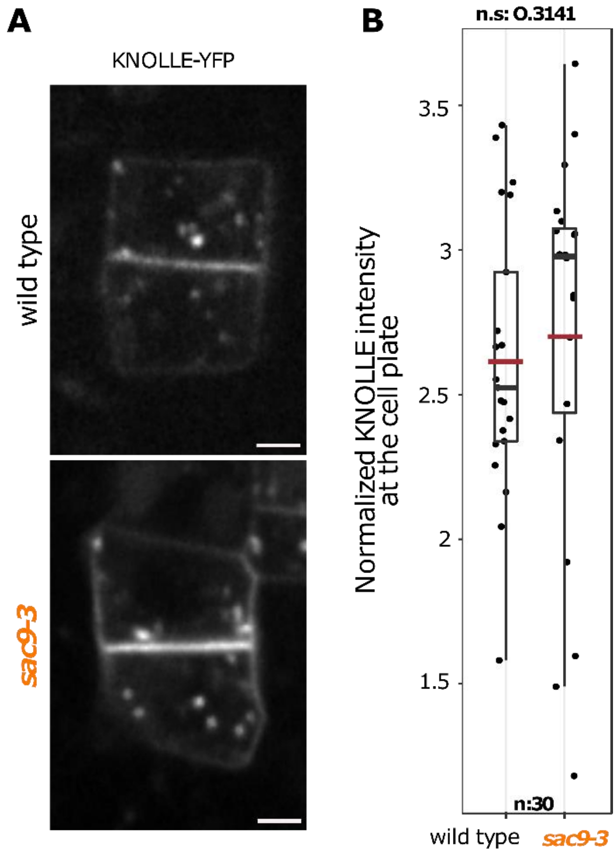


**Fig. S14 | In *sac9-3*, no dispersed vesicular localization was observed for YFP-KNOLLE.**

**A**, Confocal images of YFP-KNOLLE during the final cell plate expansion (ring and discontinuous phragmoplast) in wild type (upper panel) and *sac9-3* (bottom panel). **B**, comparison of YFP-KNOLLE intensity at the cell plate, normalized by its intracellular intensity.

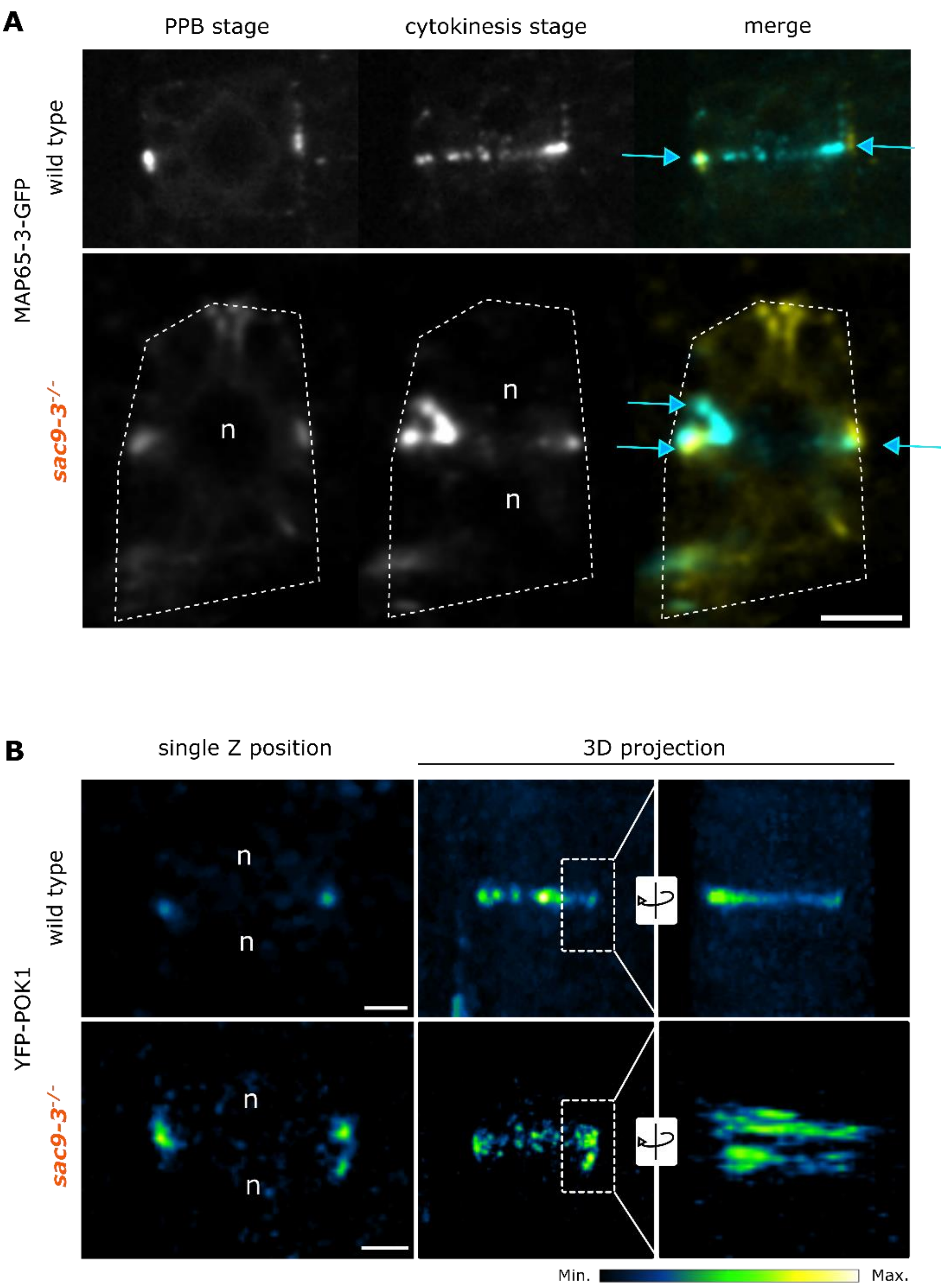


**Fig. S15 | In *sac9-3*, cell plate branching correlates with a reorganization of the cortical division zone independently of the preprophase band.**

**A**, Confocal images of the same cell expressing *MAP65-3-GFP* in wild type and *sac9-3* mutant at two different dividing steps (Pre-Prophase Band in yellow and cytokinesis in cyan). **B**, Images of YFP-POK1 in wild type and *sac9-3*. Left image represents a single optical section while the two other images are 3D projection and rotation of the cell. The signal is color coded in green fire blue (see scale bar). Scale bar, 5 µm. Blue arrow, cell plate fusion site.

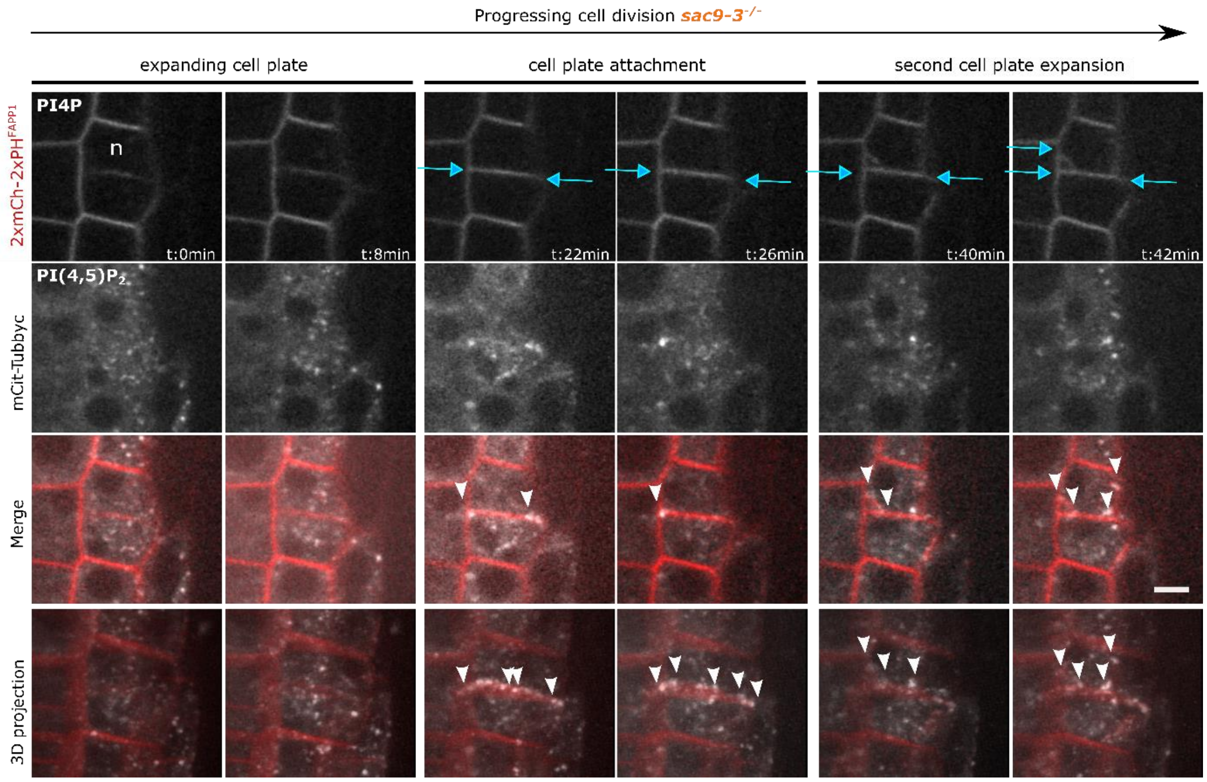


**Fig. S16 | In *sac9-3*, the PI(4,5)$P_2$ is abnormally recruited at the phragmoplast leading zone.**

Image series and 3D projection of an *in vivo* dividing *sac9-3* cells expressing both PI(4,5)$P_2$ (mCit-Tubbyc, white) and PI(4)P (2xmCh-2xPH$^{FAPP1}$, red) biosensors. Images were taken every 2 min (between 20 and 30 Z-stack per frame) for 2 h with a root tracking system (*33*). Note that here, 2xmCh-2xPH$^{FAPP1}$ is used at the membrane marker (plasma membrane and cell plate). Blue arrow, cell plate fusion site; n, the nucleus; white arrowhead, in sac9-3, PI(4,5)$P_2$ biosensor "ring" surrounding the cell plate and PI(4,5)$P_2$ biosensor spot where the branch emerged and at its tip. Scale bars, 5 µm.

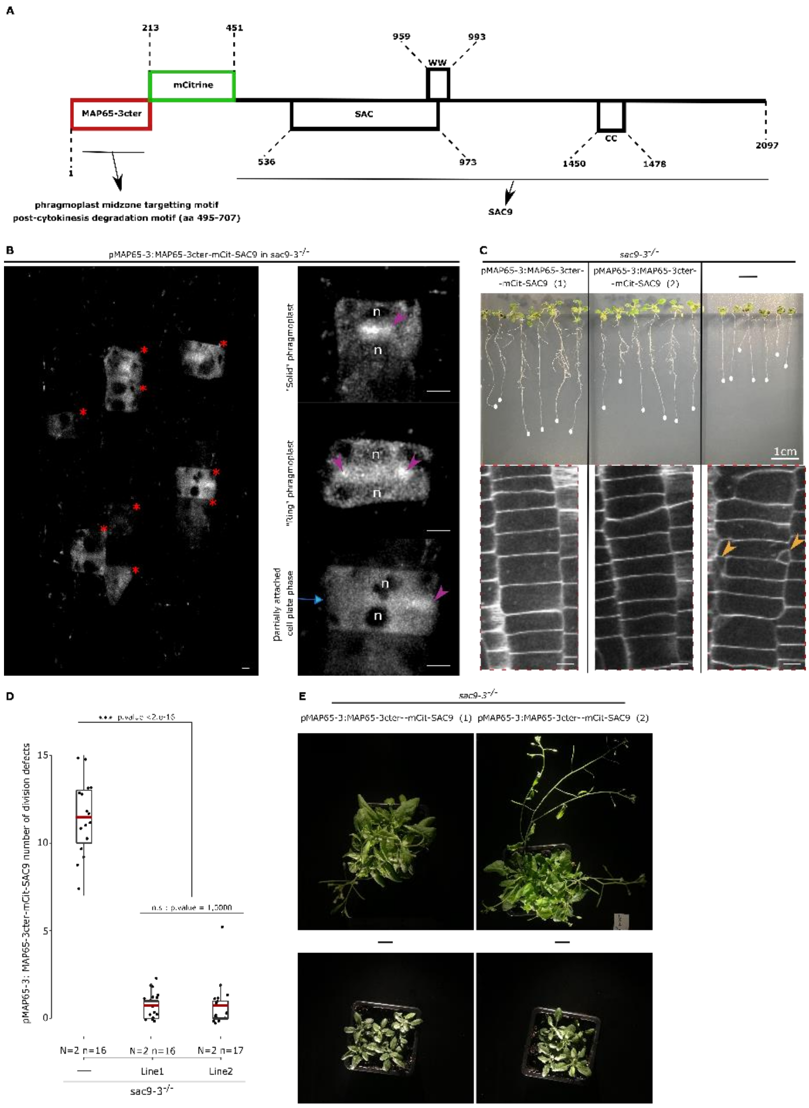


## Fig. S17 | Partial rescue of *sac9-3* when SAC9 is only present in dividing cells.

**A,** Diagram representing the 2097 amino-acid MAP65-3$^{cter}$-mCit-SAC9 protein. We fused the C-terminal (Cter) domain of MAP65-3 that contains a microtubule binding site that enables specific activity in the phragmoplast midzone and its degradation post cytokinesis (*24*) to mCIT and SAC9 genomic sequence. **B,** left: Expression of *pMAP65-3:MAP65-3$^{cter}$-mCit-SAC9* in *sac9-3* dividing cells (red star) of the root meristematic zone. Right: localization of MAP65-3$^{cter}$-mCit-SAC9 at three cytokinesis steps. Scale bars, 5µm. **C**, Phenotypes of *sac9-3* (right panel) complemented with two MAP65-3$^{cter}$-mCit-SAC9 independent lines (middle and left panels). Top panel: representative images of the macroscopic phenotype observed (21dpi); Bottom panel: images of calcofluor-stained fixed roots (10 day-old). Scale bars, 5µm. Orange arrowhead, division defects; n, nucleus; yellow arrowhead, enrichment on the cell plate; Blue arrow, the cell plate fusion site. **D**, Quantification of the number of division defects in *sac9-3* and *sac9-3* expressing *MAP65-3:MAP65-3$^{cter}$-mCit-SAC9*. In the plots, middle horizontal bars represent the median, while the bottom and top of each box represent the 25$^{th}$ and 75$^{th}$ percentiles, respectively. At most, the whiskers extend to 1.5 times the interquartile range, excluding data beyond. For range of value under 1,5 IQR, whiskers represent the range of maximum and minimum values. Results of the statistical analysis (shown in the supplementary table) are presented (N = number of replicates, n = number of cells). **E**, Representative images of the aerial phenotype observed (6-week-old plants).

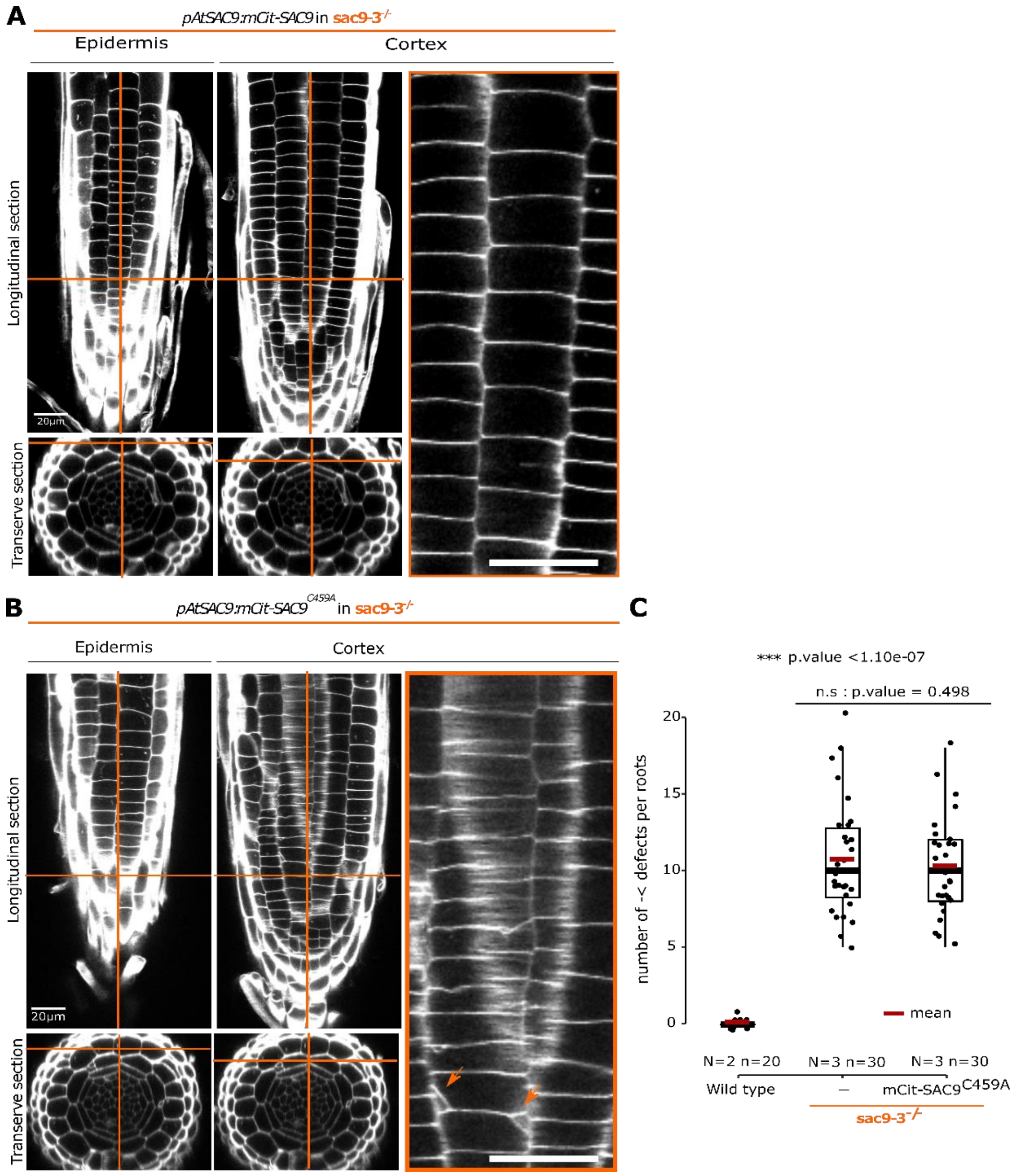


**Fig. S18 | Mutation in the putative catalytic domain of SAC9 does not rescue the phenotype during cell division.**

**A, B**, Z-stack images of calcofluor-stained fixed roots (7-day-old) *sac9-3* expressing the functional *mCit-SAC9* (A), *mCit-SAC9$^{C459A}$* (B). The left panel shows the epidermis layer in a longitudinal section (top, scale bar 20 µm.) and a transverse section (bottom). The middle panel shows the cortex layer, in the same way as the epidermis. The right panel shows a crop of the cortex layer (scale bar 5 µm). Orange arrow, cell division defects; Orange cross-section, position within the tissue for longitudinal and transverse sections. **C**, Quantification of the number of cell wall defects in wild-type, *sac9-3* and mCit-SAC9$^{C459A}$ in *sac9-3*. In the plots, middle horizontal bars represent the median, while the bottom and top of each box represent the 25$^{th}$ and 75$^{th}$ percentiles, respectively. At most, the whiskers extend to 1.5 times the interquartile range, excluding data beyond. For range of value under 1,5 IQR, whiskers represent the range of maximum and minimum values. Results of the statistical analysis (shown in the supplementary table) are presented (N = number of replicates, n = number of cells).

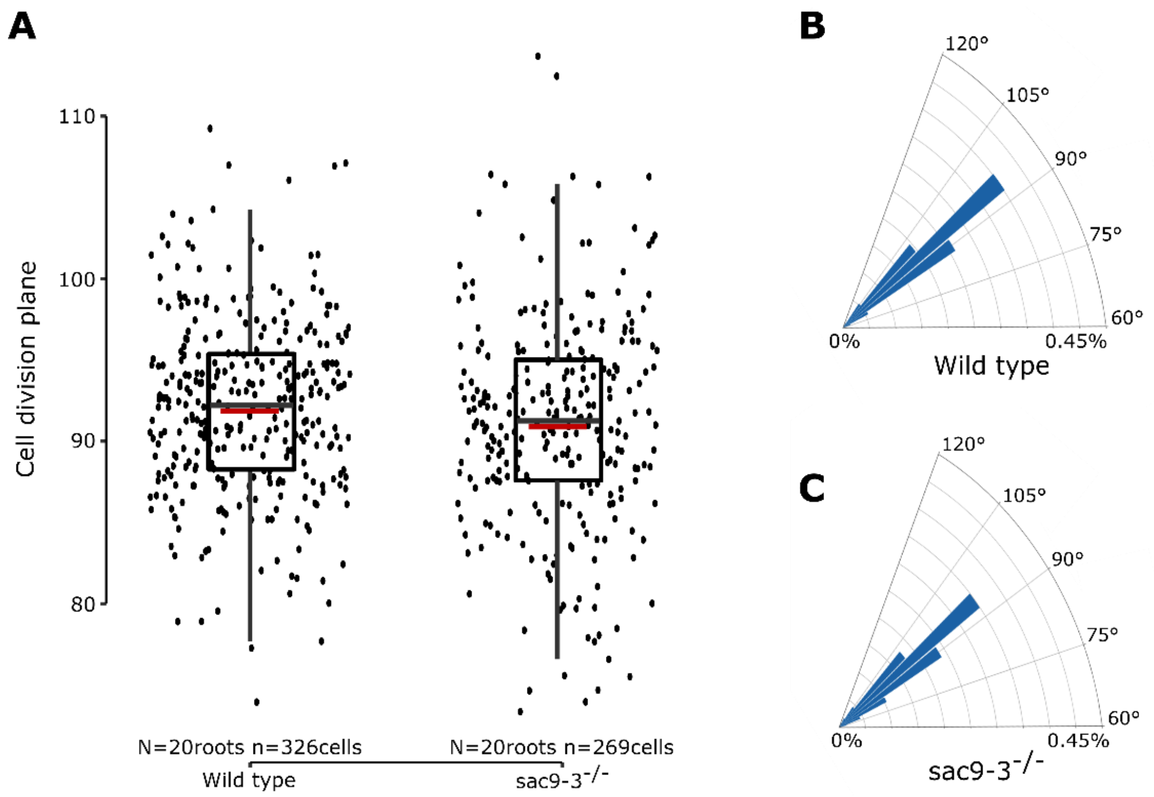


**Fig.S19 | division plane comparison between wild type and sac9-3 mutant.**

A, Comparison of division plane orientation in cortex cells (relative angle between the transverse cell wall and the root growth axis) between wild type and sac9-3 plants. In the plots, middle horizontal bars represent the median, while the bottom and top of each box represent the 25th and 75th percentiles, respectively. At most, the whiskers extend to 1.5 times the interquartile range, excluding data beyond. For range of value under 1,5 IQR, whiskers represent the range of maximum and minimum values. red bars represent the mean value. B, C, representation of cell wall angle for respectively wild type and sac9-3 plants.

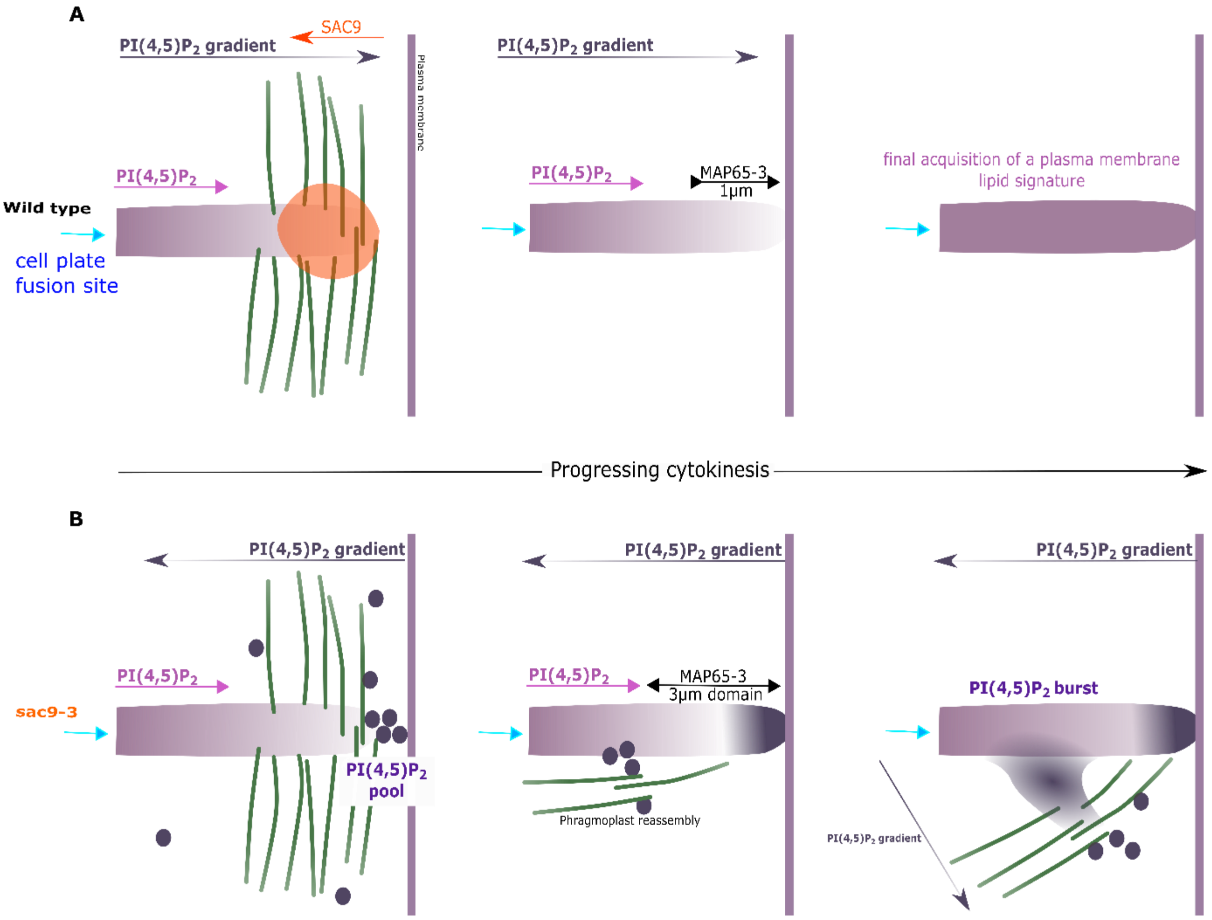


**<u>Fig. S20 | Proposed model</u>**

**A**, Concomitantly to the final cell plate attachment to the mother cell, PI(4,5)$P_2$ gradually invades the entire plasma membrane precursor from the maturing zone to the decreasing leading zone. This PI(4,5)$P_2$ patterning is tightly controlled through the action of SAC9 enzyme, directly at the cell plate or in endosomes surrounding the not yet attached cell plate leading zone. **B**, In *sac9* mutant, the abnormal accumulation of PI(4,5)$P_2$-rich endosomes close to the cell plate leading edge influences cell plate growth. Alternatively, the aberrant PI(4,5)$P_2$ patterning in sac9-3 might be perceived by the cytokinetic apparatus as in the opposite direction relative to the cell plate growth direction, creating a conflict in the spatial coordination of the expanding leading zone. In any case, mis-localized PI(4,5)$P_2$ in *sac9* lead to a MAP65-3 ~ 3 µm domain stabilization, even after cell plate complete attachment, and second to reassembly of the phragmoplast apparatus on the inner side of the MAP65-3 domain. Following phragmoplast reassembly, a cell plate protuberance emerges and expands toward the plasma membrane, with a precise geometry.

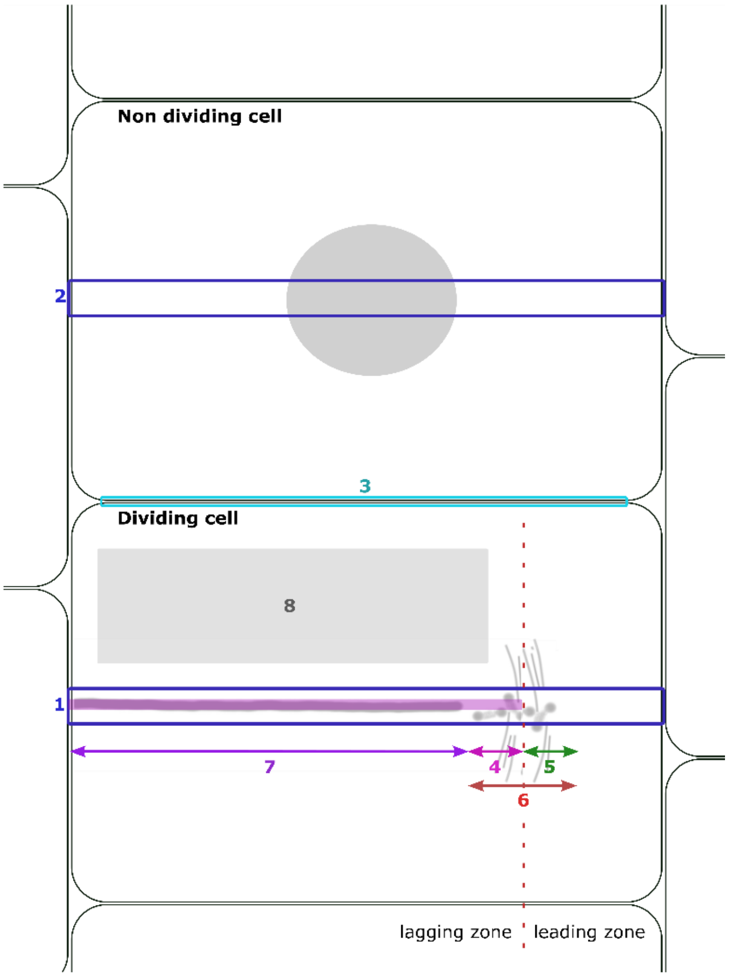


**Fig.1F, fig.S3**

$$\frac{\text{(cell plate lagging (4) or leading (5) zone intensity - cytosolic background (8))}}{\text{(phragmoplast midzone intensity (6) -cytosolic background (8))}}$$

**Fig.2C, F**

$$\frac{\text{cell plate lagging (4) or leading (5) zone intensity}}{\text{cell plate center intensit (7)}}$$

**fig.S6**

$$\frac{\text{"phragmosome" intensity of a dividing cell (1) /plasma membrane intensity (3)}}{\text{"phragmosome" intensity of a \textbf{non} dividing cell (2) /plasma membrane intensity (3)}}$$

**fig.S9**

$$\frac{\text{cell plate center intensit (7)}}{\text{plasma membrane intensity (3)}}$$

**fig.S14**

$$\frac{\text{cell plate center intensit (7)}}{\text{cytosolic background (8)}}$$

**<u>Fig. S21 | Quantification methods</u>**

Graphical representation of one dividing cell (at the discontinuous phragmoplast stage) and one non-dividing cell to illustrate the intensity quantification made on this paper. Critical zones are highlighted by arrows as well as the equations used.

# Captions for Movies S1 to S10

**Movie S1**| Colocalization analysis of tdTOM-SAC9$^{C459A}$ (magenta) with MAP65-3-GFP (green)
Time-course analysis during cytokinesis of tdTOM-SAC9$^{C459A}$ (magenta) with MAP65-3-GFP (green) using a root tracking system (*33*). The division was imaged every two minutes. Scale bar, 5µm

**Movie S2** | PI(4,5)$P_2$ behavior during cytokinesis in *sac9-3* mutant
Time-course analysis using a root tracking system (*33*) of mCit-Tubbyc (white) and 2xmCH-2xPH$^{FAPP1}$ (red) subcellular localization during cytokinesis in *sac9-3*. Here, 2xmCh-2xPH$^{FAPP1}$ is used as a marker for membranes (plasma membrane and cell plate). The division was imaged every two minutes. Scale bar: 5 µm.

**Movie S3** | MAP65-3-GFP behavior during cytokinesis in wild-type plants
Time-course analysis using a root tracking system (*33*) of MAP65-3-GFP subcellular localization during cytokinesis in WT. The division was imaged every two minutes. Scale bar : 5µm

**Movie S4 and S5** | MAP65-3-GFP behavior's during cytokinesis in *sac9-3*
Example of defective cytokinesis in *sac9-3* expressing *MAP65-3-GFP* and imaged over time using a root tracking system (*33*). The division was imaged every two minutes. scale bar: 5 µm

**Movie S6** |Mild treatment with chlorpropham induces cell plate branching
Time-course analysis using a root tracking system of the plasma membrane labeling PI4P biosensor (mCIT-P4M) in WT root treated for 2 h with chlorpropham. Here, mCIT-P4M is used as a marker for membranes (plasma membrane and cell plate). Scale bar: 10 µm

**Movie S7** | Imaging of the defects during cytokinesis in *sac9-3*
Time-course analysis using a root tracking system (*33*) of 2xmCH-2xPH$^{FAPP1}$ in *sac9-3* during cell plate defect formation (arrow). Here, 2xmCH-2xPH$^{FAPP1}$ is used as a marker for membranes (plasma membrane and cell plate). Scale bar: 5 µm

**Movie S8** | branched cell wall defects segmentation in *sac9-3*
Rotation of a branched cell wall defect (green) and its neighboring cell (gray) 3D view imaged after calcofluor staining.

**Movie S9** | branched cell wall defects segmentation in *sac9-3*
Rotation of branched cell wall defects accumulation (green) and their neighboring cell (gray) 3D view imaged after calcofluor staining.

**Movie S10** | PI(4,5)$P_2$ behavior during double cell plate attachment in *sac9-3*
Time-course analysis using a root tracking system (*33*) of mCit-Tubbyc (white) and 2xmCH-2xPH$^{FAPP1}$ (red) subcellular localization during cytokinesis in *sac9-3*. Here, 2xmCH-2xPH$^{FAPP1}$ is used as a marker for membranes (plasma membrane and cell plate). The division was imaged every two minutes. Scale bar, 5µm

# Captions for Supplementary tables S1 to S14

**Supplementary tables |** Resources and statistics.
Supplementary table S1, Reagent and resources.
Supplementary table S2, Details of the statistics corresponding to Fig. 1F.
Supplementary table S3, Details of the statistics corresponding to Fig. 2C, 2F.
Supplementary table S4, Details of the statistics corresponding to Fig. 5B.
Supplementary table S5, Details of the statistics corresponding to Fig. 6B.
Supplementary table S6, Details of the statistics corresponding to fig. S3B, S3D.
Supplementary table S7, Details of the statistics corresponding to fig. S6B.
Supplementary table S8, Details of the statistics corresponding to fig. S9B.
Supplementary table S9, Details of the statistics corresponding to fig. S10D, S10E, S10F, S10G.
Supplementary table S10, Details of the statistics corresponding to fig. S11F.
Supplementary table S11, Details of the statistics corresponding to figure S12A to S12F.
Supplementary table S12, Details of the statistics corresponding to fig. S14B.
Supplementary table S13, Details of the statistics corresponding to fig. S17D.
Supplementary table S14, Details of the statistics corresponding to fig. S18C.
